# Evaluating the effect of immunization with DNA encoding *Phlebotomus sergenti* apyrase protein (PsSP42) against *Leishmania tropica* infection in BALB/c mouse model

**DOI:** 10.1186/s13071-026-07255-x

**Published:** 2026-03-09

**Authors:** Samira Hosseinpour Jahednia, Hossein Rezvan, Hamzeh Sarvnaz, Sima Habibzadeh, Alireza Nourian, Tahereh Taheri, Negar Seyed, Elham Gholami, Sima Rafati

**Affiliations:** 1 Department of Pathobiology, Faculty of Veterinary Medicine, Bu-Ali Sina University, Hamedan, Iran; 2 Department of Immunotherapy and Leishmania Vaccine Research, Pasteur Institute of Iran, Tehran, Iran

**Keywords:** *Leishmania tropica*, Cutaneous leishmaniasis, DNA vaccine, Apyrase salivary protein, *Phlebotomus sergenti*, *Leishmania tarentolae*

## Abstract

**Background:**

*Leishmania* parasites are transmitted through the bite of infected female sand flies. The sand fly inoculum contains both the parasite and the salivary proteins, which can modulate the immune system’s function. Some of these salivary proteins have the potential to be used as a vaccine candidate. Since there have been fewer studies investigating the salivary proteins of *Phlebotomus* (*Ph.*) *sergenti*, this prompted us to select among the three protein members of *Ph. sergenti* apyrase family (PsSP40, PsSP41, and PsSP42) and measure its effectiveness as vaccine candidate against *Leishmania* (*L.*)* tropica*.

**Methods:**

To select among the three family members as the candidate for immunization, different parameters including the physicochemical characters, three-dimensional structure, virtual immune stimulatory potential, and human leukocyte antigen (HLA) class II-binding epitope content were considered. To investigate the effect of immunization with the selected antigen through immunoinformatics analysis (PsSP42) against *L. tropica* infection, we immunized BALB/c mice with two distinct recombinant plasmids (conventional VR1020 and new-generation NTC9385R) two times at 3-week intervals followed by immediate electroporation. Eight weeks post-challenge, the parasite load in draining lymph nodes was measured by quantitative real-time polymerase chain reaction (PCR). The interferon (IFN)-γ and interleukin (IL)-4 cytokines before (against recombinant *Leishmania tarentolae* expressing PsSP42) and after (against parasite frozen/thawed antigens) *L. tropica* infection (2 × 10^7^ parasite per footpad plus *Ph. sergenti* salivary gland homogenate (SGH)) were measured by enzyme-linked immunosorbent assay (ELISA).

**Results:**

On the basis of immunoinformatics analysis of three apyrase salivary proteins from *Ph. sergenti*, PsSP42 demonstrated superior HLA class II-binding peptides compared with the other two proteins (PsSP40 and PsSP41) and was selected for immunization studies. Our findings indicated that NTC-PsSP42 and not VR1020-PsSP42 plasmid immunization relatively reduced the parasite load in the draining lymph nodes. This was assigned to a significant higher IFN-γ to IL-4 ratio induced by NTC-PsSP42 immunization in comparison with pertinent controls.

**Conclusions:**

In our study, although the expected protective response was not achieved by any of the recombinant plasmids, the NTC-PsSP42 platform induced a weak Th1-polarized immune response, which partially influenced the parasite load. Since the new generation of plasmids are worth evaluating owing to the lack of antibiotic resistance genes on the backbone, we recommend further assessment of NTC-PsSP42 potential adjuvnated with immunostimulatory sequences such as as CpG motifs or even in heterologous prime-boost regimens.

**Graphical Abstract:**

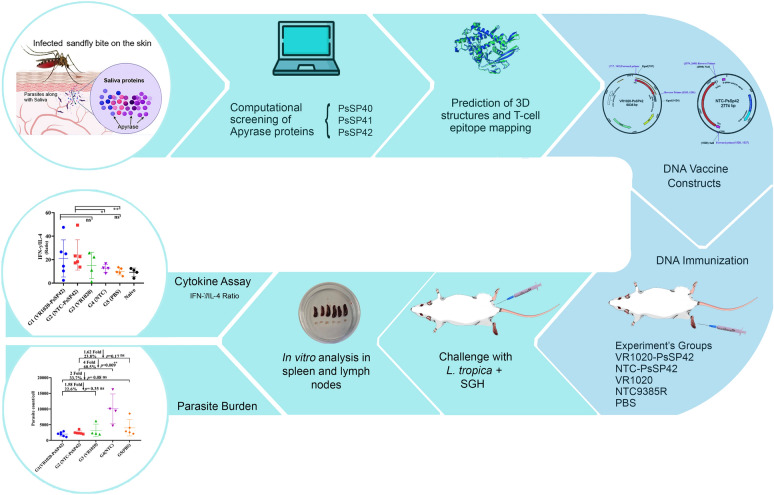

**Supplementary Information:**

The online version contains supplementary material available at 10.1186/s13071-026-07255-x.

## Background

*Leishmania* is a protozoan parasite classified under the Trypanosomatida family and Kinetoplastida order [1]. It is primarily transmitted through the bite of female sand flies of the *Phlebotomus* (Old World) and *Lutzomyia* (New World) species, which are prevalent in tropical regions [2]. Over 90 species of sand flies have been identified as vectors of human leishmaniasis, which causes three main forms of leishmaniasis: cutaneous leishmaniasis (CL), mucocutaneous leishmaniasis (MCL), and visceral leishmaniasis (VL) [3]. CL is the most prevalent form of the disease, causing skin lesions, ulcers, and scars with significant social and psychological impacts. According to the latest information by the World Health Organization (WHO), between 600,000 and 1,000,000 people are infected with CL annually, most of them living in the Americas, the Mediterranean Basin, the Middle East, and Central Asia, including Iran [64].

Two distinct forms of CL occur in Iran: zoonotic leishmaniasis (caused by *Leishmania major* and most predominantly transmitted by *Ph. papatasi* sand fly) and anthroponotic leishmaniasis (caused by *Leishmania tropica* and most predominantly transmitted by *Ph. sergenti*) [4]. Given the growing concerns over the drug resistance and toxic side effects of current treatments, developing an effective vaccine against leishmaniasis has become a critical necessity [5, 6].

Over the past four decades, vaccine research against leishmaniasis has considered not only parasite-derived antigens but also sand fly salivary molecules. During blood feeding, sand flies inoculate bioactive components of saliva into the host skin, which modulate the host’s hemostatic system [7], including vasodilation (maxadilan in *Lutzomyia longipalpis* [8] and adenosine in *Ph. papatasi* [9]), platelet aggregation (apyrase in *Ph. papatasi* [10]), or tissue permeability enhancement (hyaluronidase in *Phlebotomus tobbi* and *Phlebotomus sergenti* [11]). These salivary factors facilitate successful blood acquisition by the vector; however, there is ample evidence to indicate that they are evolutionarily responsible for enhancing pathology and infection severity [12]. Meanwhile, prior exposure to non-infectious bites [13] or immunization with specific components [14] results in elicitation of a Th1 response to salivary antigens and protects against the parasite. This has implications for protective vaccination where we need a rapid (within hours) and robust (IFN-γ) Th1 immune response early after sand fly bites [15].

Among all the salivary proteins, apyrase is found in many bloodsuckers and prevents blood clotting [17]. These calcium-magnesium dependent anti-homeostatic enzymes hydrolyze both adenosine triphosphate (ATP) and adenosine diphosphate (ADP) to adenosine monophosphate (AMP), hence preventing platelet aggregation following tissue damage or injury to blood vessels. In addition to their hydrolytic activity, sand fly apyrases are highly antigenic, contributing to their immunological relevance [17, 16]. The antigenic properties of sand fly salivary apyrase proteins have been reported in several studies, including works on *Ph. ariasi* [17] or *Ph. argentipes* in hamsters [18, 19], *Ph. orientalis* in domestic animals [16], *Ph. perniciosus* in dogs [19, 20], and *Ph. kandelakii* in BALB/c mice [21]. However, the most direct evidence is generated from analysis of human peripheral blood mononuclear cells (PBMCs) stimulated with *Ph. papatasi* apyrase to proliferate and secret IFN-γ [22]. The fact that apyrase is conserved across diverse sand fly vectors is an added value for apyrase as a broad-spectrum vaccine component or as a component of a multivalent vaccine [23].

Given the limited amount of research on *Ph. sergenti* salivary components, this study was undertaken to assess the efficacy of apyrase from *Ph. sergenti* as a vaccine candidate. The apyrase family of *Ph. sergenti* has three members, including PsSP40, PsSP41, and PsSP42. After selecting PsSP42 as the potential candidate among the three family members on the basis of immunoinformatics results and the previous study by Gholami et al. [24] regarding the immunogenicity of apyrase family proteins (without any preference among the three), two distinct DNA vaccine constructs, namely VR1020-PsSP42 (a conventional plasmid) and NTC-PsSP42 (a new-generation plasmid), were prepared. In contrast to conventional plasmids such as VR1020, NTC9385R (nanoplasmid) has a reduced backbone size and lacks antibiotic resistance markers, which is an important safety consideration in DNA-based vaccines for humans. Although a potential protective response was not achieved by any of the recombinant plasmids, the NTC-PsSP42 platform induced a weak Th1-polarized immune response, which partially influenced the parasite load. Considering the positive potential of new-generation versus conventional plasmids, it is recommended to examine the protective effect of NTC-PsSP42 complemented with adjuvants such as CpG motifs, prime-boost regimens, or polytope-based vaccines with protein-specific epitopes of apyrase family members.

## Materials and methods

### Reagents

All solutions were prepared using apyrogenic deionized water (MilliQSystem, Millipore, Molshem, France). Real QPlus 2 × master mix green high Rox was purchased from Ampliqon (Denmark). Endo-Free Plasmid Mega kit, RNeasy Mini Kit, and anti-His antibody (Penta His Antibody, (Cat. No. 34660, Mouse IgG1 isotype) were purchased from QIAGEN, (Germany). Anti-Mouse IgG (Fc specific) peroxidase (cat. no. A0168) was purchased from Sigma-Aldrich (Germany). Sodium dodecyl sulfate (SDS), Tris–HCL, Tris-base, Panceau-S, and cell culture reagents including Schneider’s Insect Medium (SIM), Dulbecco’s modified Eagle medium (DMEM) medium, 4-(2-hydroxyethyl)-1-piperazineethanesulfonic acid (HEPES), l-glutamine, adenosine, and hemin were supplied by Sigma, (Germany). Bovine serum albumin (BSA) and diaminobenzidine (DAB) were supplied by Merck, (Germany). G418 antibiotic and fetal calf serum (FCS) were provided through Gibco (Life Technologies GmbH, Germany). Protran nitrocellulose transfer membrane was from Schleicher and Schuell BioScience (Germany). All necessary materials for PCR including enzymatic digestion, and gel electrophoresis were prepared by Roche (Germany). Specific primers for amplification of the target genes were synthesized by Metabion (Germany). Cytokine kits (IFN-γ and IL-4) were purchased from DuoSet R & D (USA). The bicinchoninic acid (BCA) protein assay kit was prepared by Thermo Fisher Scientific (USA). Peroxidase substrate for ELISA systems was provided by KPL (ABTS, USA). Mammalian tissue DNA extraction kit was purchased from Viragen (Iran). NTC9385R-MCS was from Nature Technology Corporation (NTC), USA. The salivary gland homogenate (SGH) and VR1020, and VR1020-PsSP42 plasmids were received as a gift from Dr. Jesus G. Valenzuela (Vector Molecular Biology Section, Laboratory of Malaria and Vector Research, National Institute of Allergy and Infectious Diseases, National Institutes of Health, Rockville, MD, USA). pLEXSY-neo2 vector (cat. no. EGE-233) was provided by Jena Bioscience (Germany).

### Amino acid sequence retrieval and alignment

The mammalian codon optimized nucleotides encoding full open reading frames of the three apyrase proteins from *Ph. sergenti* saliva (PsSP40: HM560860.1, PsSP41: HM560862.1, and PsSP42: HM560861.1) were received as a gift in VR1020-TOPO plasmid. The secretory signal peptide of tissue plasminogen activator (replaced for the original sand fly specific-secretory signal peptide) and 6 histidine tag (His-tag) were inserted at the upstream and downstream of the target gene, respectively. Protein sequences were then aligned by using the CLUSTALW (https://www.genome.jp/tools-bin/clustalw) server, and the similarity between sequences was recorded in percent [25].

### Physicochemical, antigenicity, and allergenicity properties of apyrase proteins

The major physicochemical characteristics of the three proteins including molecular weight, theoretical isoelectric point (PI), instability index, and grand average of hydropathicity (GRAVY) were evaluated through ProtParam (https://web.expasy.org/protparam) [65]. The GRAVY score typically ranges from −2 to +2 for most proteins. Proteins with negative GRAVY score are hydrophilic. The instability index approximates the protein’s stability when placed in a test tube. For stable proteins, the instability index is lower than 40. Antigenicity of the proteins was evaluated by ANTIGENpro (https://scratch.proteomics.ics.uci.edu/https://scratch.proteomics.ics.uci.edu/explanation.html#ANTIGENpro) [26]. Proteins scoring over 0.5 were categorized as antigen. To further estimate the allergenicity of proteins, two servers including Allergenfp v1.0 (https://ddg-pharmfac.net/AllergenFP/https://ddg-pharmfac.net/AllergenFP/) and Allertop v2.0 (https://www.ddg-pharmfac.net/AllerTOP) were utilized [27].

### Protein structure prediction

The sequence of all three apyrase proteins was submitted to I-TASSER (https://zhanggroup.org/I-TASSER/) server [28] to obtain the three-dimensional (3D) protein structures. The software constructs 3D models from the amino acid sequence utilizing iterative threading assembly simulation. The validity of models was measured by confidence score (C-score), which ranges between −5 and 2. A more positive C-score indicates a model with greater confidence.

### Virtual immune simulation

The immune responses after immunization with each individual apyrase protein were predicted by C-ImmSim server (https://kraken.iac.rm.cnr.it/C-IMMSIM/) [29]. C-ImmSim is an agent-based model to identify the immunogenicity of proteins using position-specific scoring matrices (PSSM). This server predicts cellular and humoral immune responses induced by an antigen in humans with HLA alleles of interest. Immunization was simulated at time steps 1 and 63, with 3-week intervals. Each time step is equal to 8 h, reflecting cell division cycle in real environment. Thus, the first immunization was set on time step 1 at day 0, and the second immunization on time step 63 at day 21. All simulation parameters were kept as default except for the HLA alleles, which were set on the most frequent alleles in Iranian population, including HLA-DRB1*11:01 and HLA-DRB1*15:01. T helper cell polarization, diversity, and cytokine production were compared after immunization with each individual protein. The Simpson or D-index was referred to as a measure of diversity. Lower Simpson index value (closer to zero) indicates increased diversity and a higher prevalence of various dominant T-cell clones.

### Screening and prediction of HLA class II-binding epitopes based on frequent alleles in Iranian population

First, the frequencies of HLA class II alleles in Iranian population were extracted from Royan Cord Blood Bank Data (*n* = 15,600) deposited in the Allele Frequency Net Database (http://www.allelefrequencies.net) [30]. Six most frequent HLA class II alleles with a frequency over 15% were selected. Then, the three protein sequences were screened individually using four different HLA class II prediction tools, namely IEDB (http://tools.iedb.org/mhcii/) [31], http://tools.iedb.org/main/tcell/, SYFPEITHI (http://www.syfpeithi.de/0-Home.htm, http://www.syfpeithi.de/bin/MHCServer.dll/EpitopePrediction.htm) [32], NetMHCII-2.3 (https://services.healthtech.dtu.dk/services/NetMHCII-2.3/), and Rankpep (http://imed.med.ucm.es/Tools/rankpep.html) [33], each working with a different algorithm. “IEDB recommended method” was chosen for peptide prediction by IEDB, utilizing a reference set of 27 human alleles (complete human leukocyte antigen reference set with population coverage exceeding 97%). Epitope selection relied on percentile rank calculation (where a lower percentile rank indicates higher affinity). Peptides with percentile rank below 5% were selected as binders. SYFPEITHI is a matrix-based approach, and peptides scoring over 20 were screened as binder. Artificial neural network (ANN)-based NetMHCII-2.3 screens epitopes on the basis of half-maximum inhibitory concentration (IC_50_) value. Peptides with high binding affinity have an IC_50_ value below 50 nM, being marked as strong binders, while weak binders scored below 500 nM. Rankpep is a server that utilizes position specific scoring matrices (PSSMs) related to a specific binding threshold (PSBT). Epitopes scored above this threshold are screened as binder. The sequences were also analyzed for peptides binding to mouse IA and IE alleles, as well. The same criteria were applied using the same servers as described before.

### Prediction of IFN-γ inducing peptides

Since the generation and secretion of IFN-γ by MHC-II activated CD4^+^ T-helper cells plays an important role in fighting against leishmaniasis, IFNepitope server (https://webs.iiitd.edu.in/raghava/ifnepitope/predict.php) was used to identify peptides from the apyrase proteins with the potency of IFN-γ induction [34]. All selected epitopes in previous step were individually analyzed for IFN-γ induction. The selected threshold was set on 0.5 as IFN-γ production determinant.

### Preparation of DNA constructs

On the basis of the immunoinformatics criteria, PsSP42 was selected as a promising immunogenic protein. The complete open reading frame of PsSP42 gene was amplified from VR1020-PsSP42 using Taq DNA polymerase and PsSP42-specific primers (PsSP42-forward: 5′-GCGTCGACATGGATGCAATGAAGAGAGGGCT-3′ and PsSP42-Reverse: 5′-TAGCGGCCGCACAGCAGATCTGGATCGA-3′, with *Sal*I and* Not*I restriction sites, respectively (underlined). Then, the PCR-amplified PsSP42 gene was subcloned into *Sal*I and *Not*I sites of NTC 9385R-MCS (NTC) to produce NTC-PsSP42. After sequence confirmation, large scale purification of VR1020, NTC, VR1020-PsSP42, and NTC-PsSP42 plasmids was performed via ion-exchange chromatography with an Endotoxin-free plasmid Giga kit according to the manufacturer’s instructions for further use in mice immunizations. The PsSP42 sequence was also subcloned into *Sal*I and *Not*I restriction sites of the pLEXSY-neo2 vector. The pLEXSY-PsSP42 was then transiently transfected into *L. tarentolae*.

### Generation of recombinant *L. tarentolae* expressing PsSP42 through episomal transfection

*L. tarentolae* (Tar II, ATCC 30267) strain was cultured at pH 7.2 and 26 °C in SIM supplemented with 5% heat-inactivated FCS (hi-FCS), 0.5 μg/ml hemin, and 50 μg/ml gentamicin. According to the preset transfection protocol [35], 1.3 × 10^8^ log-phase parasites were washed and resuspended in 300 μl of ice-cold electroporation buffer (21 mM HEPES, 137 mM NaCl, 5 mM KCl, 0.7 mM Na_2_HPO_4_, 6 mM glucose, pH 7.5) and mixed with 20 μg of pLEXSY-PsSP42 DNA. Cells were electroporated with two pulses of 450 V, and 450 μF using a Bio-Rad Gene Pulser Ecell device (Bio-Rad, USA). Electroporated parasites were incubated in antibiotic-free SIM containing 5% hi-FCS at 26 °C for 24 h, then transferred to a 24-well plate containing 80 μg/ml G418. Antibiotic-resistant parasites were then selected within 2 weeks. Recombinant *L. tarentolae* expressing PsSP42 (*L. tarentolae*-PsSP42) was further confirmed by PCR amplification on genomic DNA as template using PsSP42-specific primers to amplify the 1100-bp fragment. The expression of PsSP42 in the recombinant parasites was confirmed by Western blot analysis using anti-His antibody according to the previously described protocol [36].

### Parasite culture and antigen preparation

For infectious challenge, the popliteal lymph nodes from already infected BALB/c mice with* L. tropica* (MOHM/IR/09/Khamesipour-Mashhad) were isolated and cultured in SIM supplemented with 10% hi-FCS 0.5 µg/ml of hemin, and 100 µg/ml of gentamicin. Five days post culture, promastigote parasites were collected, washed, and resuspended in PBS buffer (8 mM Na_2_HPO_4_, 1.75 mM KH_2_PO_4_, 0.25 mM KCl, and 137 mM NaCl) for mice challenge.

For antigen preparation, *L. tarentolae*-PsSP42 and also *L. tropica* promastigotes at stationary phase were washed in PBS then embedded in liquid nitrogen for a few seconds and immediately thawed in 37 °C water bath. This process was repeated until no intact parasite was discriminated under the light microscope. The concentration of frozen/thawed (F/T) antigens was quantified by BCA method according to the manufacturer’s protocol, and the antigens were kept at −80 °C until use.

### Immunization and infectious challenge

To evaluate the immunogenic characteristics of two different DNA plasmids that encode for *Ph. sergenti* apyrase salivary PsSP42, BALB/c mice were divided into five groups (G1 to G5). Ten (G3, G4, and G5) to 12 mice (G1 and G2) per group (along with 8 naïve mice) were first anesthetized by ketamine (1%)/xylazine (0.05%) intraperitoneal injection [37]. All mice (except for naïve group) were subcutaneously immunized in the left footpad with either 50 μl (1 μg/μl) of recombinant DNA plasmids including VR1020-PsSP42 (in G1) and NTC-PsSP42 (in G2) or 50 μl (1 μg/μl) of the control NTC9385R (in G3) and VR1020 plasmids (in G4) respectively and PBS (in G5) two times at 3-week intervals. DNA immunizations were followed by immediate electroporation using BTX HARVARD APPARATUS-ECM 830, (USA) with footpad applicable electrodes. After fixing the injected footpad between the electrodes, six pulses of 60 V, 20 ms each, with 200 ms intervals were applied [38]. Three weeks after the last immunization, BALB/c mice in different groups were challenged with 2 × 10^7^ late stationary phase *L. tropica* plus *Ph. sergenti* SGH (0.5 pair salivary gland homogenate/mouse according to standard protocols [39–41]) at the right footpad (Table 1).

**Table 1:** Immunization regimes in BALB/c mouse groups

Group	First immunization	Second immunization	Infectious challenge
G1	VR1020-PsSP42	VR1020-PsSP42	Late stationary phase *L. tropica* plus *Ph. sergenti* SGH
G2	NTC-PsSP42	NTC-PsSP42	
G3	VR1020	VR1020	
G4	NTC-9385R-MCS	NTC-9385R-MCS	
G5	PBS	PBS	
Naïve	None	None	None

### Cytokine profile analysis

The cytokine production levels in the immunized (G1 and G2) control and naïve groups (G3, G4, and G5) were measured before and 8 weeks post *L. tropica* + SGH challenge. Mice from each group were ethically euthanized. The spleens were excised in sterile conditions and homogenized followed by removal of erythrocytes using ACK lysis buffer (0.15 M NH_4_Cl, 1 m M KHCO_3_, and 0.1 mM Na_2_EDTA). The splenocytes were then washed and resuspended in a complete phenol red-free DMEM medium supplemented with 10% hi-FCS, 1% Ll-glutamine, 1% HEPES, 0.1% 2ME, and 0.1% gentamicin. To determine the concentration of cytokines, 3.5 × 10^6^ cells were seeded in 48-well plates with various antigens including *L. tropica* F/T antigen (10 µg/ml), F/T antigen of *L. tarentolae*-PsSP42 for before challenge stimulation (20 µg/ml) or concanavalin A (5 µg/ml). The plates were incubated at 37 °C with 5% CO_2_. Culture supernatants were collected after 3 and 5 days to measure the IL-4 and IFN-γ production, respectively. The concentration of cytokine production was measured using a Sandwich Elisa Kit according to the manufacturer’s protocol.

### Quantification of lymph node parasite burden using quantitative real-time PCR

Eight weeks after the infectious challenge, quantitative real-time polymerase chain reaction (qRT-PCR) was used to determine the extent of parasite propagation in the draining popliteal lymph nodes (LNs). The genomic DNA was extracted from each homogenized LN using the Viragen DNA extraction kit according to the manufacturer's instructions. The DNA concentration was then measured by Nanodrop spectrophotometer (ND 1000, USA). qRT-PCR was run according to the preset protocols [35, 45]. Briefly kinetoplastid DNA of *L. tropica* was amplified by KDNA1F (5′-GGGTAGGGGCGTTCTGC-3′) and KDNA1R (5′-TACACCAACCCCCAGTTTGC-3′) primers. Mouse glyceraldehyde 3-phosphate dehydrogenase (GAPDH) forward (5′-CGTCCCGTAGACAAAATGGT-3′) and reverse (5′-TTGATGGCAACAATCTTCAC-3′) primers were used for GAPDH gene amplification to normalize the parasite load [35].

For qRT-PCR-based absolute quantification of *L. tropica* parasite in each individual lymph node by Step One Plus Real-time PCR device, 80 ng of genomic DNA was used in a total volume of 10 µl. A standard curve was drawn using tenfold diluted *L. tropica* genomic DNA, ranging from 2 × 10^8^ to 2 × 10^1^ parasites. A reference gene standard curve using fivefold dilution of GAPDH was calculated in the range of 2.7 × 10^4^ to 1 × 10^2^ DNA copies from naïve lymph node. The PCR program was set at 95 °C for 15 min, followed by 45 cycles at 95 °C for 15 s, and at 60 °C for 1 min using Ampliqon Syber Green Master Mix (High ROX). After extracting both kDNA and GAPDH copy numbers on related standard curves, the parasite burden per cell was determined using the following formula: parasite count per cell = parasite copy number $\div$ (copy number of GAPDH $\div$ 2). This way, the parasite copy number was normalized proportional to every single GAPDH copy number (which represents a cell) [35].

### Ethics statement

Female BALB/c mice (ranging 6–8 weeks of age) in this research were obtained from Pasteur Institute of Iran. All animal experiments were conducted by the Institutional Animal Care and Research Advisory Ethics Committee of the Pasteur Institute of Iran (IR.RII.REC.1400.076.) in complete compliance with the Particular National Ethical Framework for Biomedical Research (MOHME-2005).

### Statistical analysis

Prism 8.0 GraphPad was utilized for statistical analysis. Based on data passing normality by Shapro-Wilk test, Student’s *t*-test was used for analysis (the *p-*values below 0.05 being considered as significant). The analysis was first run inside each immunization group, then the two immunizations were compared on the basis of the results obtained inside each immunization. The presented data are representative of two independent experiments just for parasite burden measurement.

## Results

### Comparing *Ph. sergenti* apyrase family members in terms of physicochemical, structural, and immunological simulation properties

The retrieved sequences of the three apyrase proteins were aligned together (Additional file 1, Fig. S1) using CLUSTALW server. The sequence similarities were calculated as 88.89, 91.19, and 89.83 between SP40 and SP41, SP40 and SP42, and SP41 and SP42, respectively. Obviously, the three proteins are closely related in sequence, with more similarity between PsSP40 and PsSP42. This sequence similarity was confirmed by protein 3D structure predicted by I-TASSER server (Additional file 2, Fig. S2). As illustrated, the three proteins have C-scores close to the upper limit of the accepted range (−5 to 2), which ensures an acceptable 3D structure similar to the actual structure. As predicted by the server, all three proteins are functionally close to a human apyrase molecule with hydrolase activity (PDB ID: 1S1D). Although PsSP41 is shorter in length compared with PsSP40 and PsSP42 (Additional file 1, Fig. S1), but structurally and functionally it is similar to the other two. This modeling further confirmed that all the three proteins are closely related in structure and function with very similar scores. ProtParam tool was then used to determine the physicochemical properties including theoretical PI, molecular weight, average of hydropathicity (GRAVY) and instability index of the three apyrase protein sequences. All three proteins are stable and hydrophilic with instability index < 40 and negative GRAVY (Additional file 3, Table S1). According to the AllergenFP, all three proteins were determined as non-allergen; however, the AllerTop server confirmed this result only for the PsSP41 and PsSP42 proteins. Meanwhile, according to ANTIGENpro results, all three apyrases are probably antigenic for the immune system (Additional file 4, Table S2). Ultimately, the immune simulations of the three vaccine candidates of interest were compared in silico using the C-ImmSim online tool (Additional file 5, Fig. S3). As indicated in column A, considerable amounts of IL-12 (light-blue line) are produced in response to all three candidates, which results in the production of significant amounts of IFN-γ (purple line). Instead, IL-10 (black line) and TGF-β (orange line) as anti-inflammatory cytokines are much less induced compared with IFN-γ. Furthermore, the inset plots of all three candidates (column A, the upper right plots in each graph) indicate a high level of IL-2 (orange line), particularly following the second dose of immunization on day 21, as further evidence of T-cell proliferation. Of note, the Simpson index (*D*) for clonal specificity investigation (close to zero) indicates possible diversity in immune responses induced by all three apyrases, which is reflected in the differentiation and diversity of CD4^+^T cell population. As indicated in column B (a, c, e), following the second immunization with the individual proteins, the level of active and duplicating T helper cells increases dramatically, being mainly of Th1 type (almost 90%) and not Th2 and Th17 (almost 0%). Together, these findings predict a Th1 deviation following two immunizations as indicated in column B (b, d, f) with a similar pattern observed for all three proteins. Generally speaking, the in silico comparison of all three apyrase antigens indicated very similar conformational and immunological properties despite subtle sequence differences among the three antigens. Therefore, to select one candidate, we relied on the HLA class II-binding epitope content of each protein.

### HLA class II-binding epitope content of apyrase proteins

**Fig. 1:**
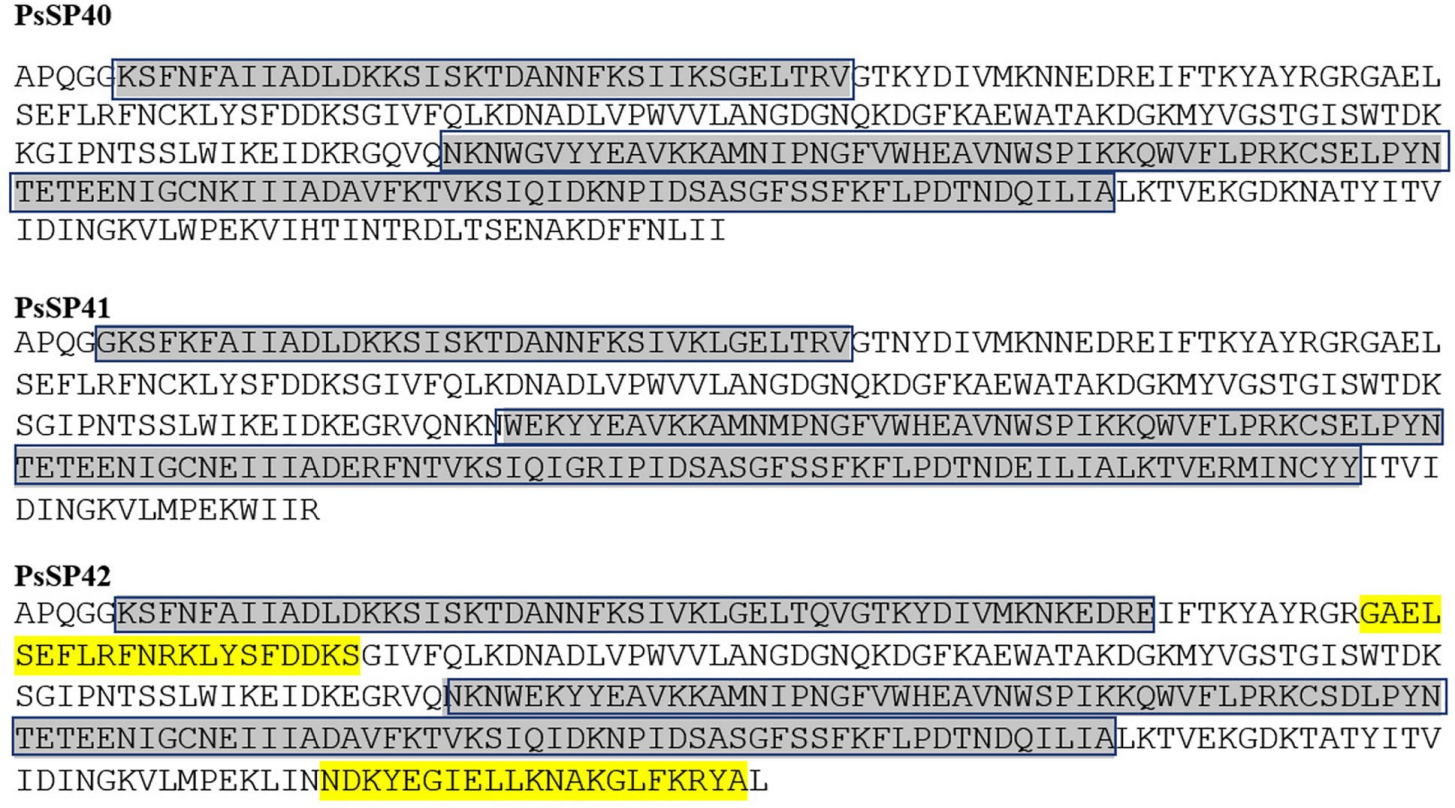
Regions on all three apyrase proteins (PsSP40, PsSP41, and PsSP42) where most of the peptides are derived from (gray highlights). Yellow highlights indicate two regions where additional SP42-specific peptides are derived

Leishmaniasis control is professionally mediated by Th1 cells. Therefore, one important criterion for a protein to be a good vaccine candidate could be the quantity and quality of the MHC class II peptides presented to CD4^+^ T cells, which is referred to as fishing for antigens using epitopes as bait. In this respect, we compared the three apyrase proteins for their MHC class II-binding peptide content depending on the most frequent HLA alleles in Iranian population. According to the Cord blood data bank in Allele Frequency Net data bank, six top frequent alleles in Iranian population included HLA-DRB1*03:01 (16.2%), HLA-DRB1*04:01 (19.7%), HLA-DR-B1*07:01 (16.9%), HLA-DRB1*11:01 (32.4%), HLA-DRB1*13:01 (18.5%), and HLA-DR-B1*15:01 (20.4%). Different tools with distinct algorithms were included for prediction, and 15-mer peptides were selected only if they passed the thresholds of at least two different prediction tools. While analyzing human HLA class II-binding peptides (presented in the top six HLA alleles in Iranian population), we detected two regions on all three proteins (gray highlights in Fig. 1) where most of the peptides are derived from. Furthermore, we have detected two separate regions on PsSP42 protein where most of the PsSP42 specific peptides are derived from (yellow highlights in Fig. 1). Despite a very similar sequence, PsSP42 has 61 HLA class II restricted peptides, among which 33 peptides are specific to this protein in comparison with the other two apyrases (PsSP40 and PsSP41). Fourteen peptides out of 33 are derived from two regions related to PsSP42 sequence (yellow highlights in Fig. 1). Furthermore, many specific peptides derived from PsSP42 are presented in more than one HLA allele (11 peptides), prioritizing this proteins ove the others with respect to population coverage. Twenty-five specific epitopes were predicted in HLA-DRB1*11:01, which has the highest allele frequency among Iranians, 12 of which theoretically induce IFN-γ (Table 2). In comparison, PsSP40 which is closer to PsSP42 in sequence, has 45 HLA class II restricted peptides, 12 of which are specific and 9 out of 12 induce IFN-γ production as predicted (Table 3). Only six specific peptides are presented in HLA-DRB1*11:01. Owing to the greater sequence similarity with PsSP42, PsSP40 was ranked after PsSP42 as vaccine candidate, considering the HLA class II results. Finally, the PsSP41 results highlighted 30 peptides (Table 4), many of which are PsSP41 specific but presented in one HLA allele, and very few of which have IFN-γ induction ability (Table 4). To further select between PsSP40 and PsSP42, mouse MHC class-II binding peptides were analyzed as well. As indicated in Table 5, PsSP42 resulted in eight peptides, six of which induced IFN-γ as predicted. However, PsSP40 resulted in six peptides, while four out of six could induce IFN-γ production. PsSP41 however resulted in only two peptides with IFN-γ induction potential. The epitope prediction by immunoinformatics analysis based on mouse MHC prediction barely returns impressive results. Since the prediction tools are mainly trained by human HLA presented peptides and not mouse MHC, the epitope prediction by the same procedure implemented for human HLA in this study resulted in very few epitopes in mouse. As a result, the in vivo evaluation of vaccine efficacy was continued using PsSP42 as the first-ranked candidate determined by human HLA presented epitopes. The pipeline for comparing the T-cell epitope content of antigen candidates together and selecting among them was already introduced by other studies as fishing for antigen using epitopes as bait [42].

**Table 2:** HLA class II restricted specific and nonspecific epitopes derived from PsSP42 protein

No.	Peptide name	HLA specificity	IFN-γ induction	Specific/nonspecific
KSFNFAIIADLDKKSISKTDANNFKSIVKLGELTQVGTKYDIVMKNKEDRE				
1	KSFNFAIIADLDKKS	HLADRB1*:03:01	Neg	Nonspecific
2	SFNFAIIADLDKKSI	HLADRB1*:03:01 HLADRB1*:04:01	Neg	Nonspecific
3	FNFAIIADLDKKSIS	HLADRB1*:03:01	Neg	Nonspecific
4	NFAIIADLDKKSISK	HLADRB1*:03:01 HLADRB1*:04:01	Neg	Nonspecific
5	FAIIADLDKKSISKT	HLADRB1*:03:01 HLADRB1*:11:01	Neg	Nonspecific
6	AIIADLDKKSISKTD	HLADRB1*:03:01	Neg	Nonspecific
7	KTDANNFKSIVKLGE	HLADRB1*:11:01 HLADRB1*:15:01	Pos	Nonspecific
8	TDANNFKSIVKLGEL	HLADRB1*:11:01	Neg	Nonspecific
9	DANNFKSIVKLGELT	HLADRB1*:11:01	Neg	Nonspecific
10	ANNFKSIVKLGELTQ	HLADRB1*:11:01	Pos	Specific
11	NNFKSIVKLGELTQV	HLADRB1*:11:01	Pos	Specific
12	NFKSIVKLGELTQVG	HLADRB1*:11:01	Neg	Specific
13	FKSIVKLGELTQVGT	HLADRB1*:11:01	Neg	Specific
14	GTKYDIVMKNKEDRE	HLADRB1*:11:01	Pos	Specific
15	KKSISKTDANNFKSI	HLADRB1*:03:01 HLADRB1*:07:01	POS	Nonspecific
GAELSEFLRFNRKLYSFDDKS				
16	GAELSEFLRFNRKLY	HLADRB1*:11:01 HLADRB1*:15:01	Neg	Specific
17	AELSEFLRFNRKLYS	HLADRB1*:03:01 HLADRB1*:11:01 HLADRB1*:13:01	Neg	Specific
18	ELSEFLRFNRKLYSF	HLADRB1*:03:01 HLADRB1*:13:01	Neg	Specific
19	LSEFLRFNRKLYSFD	HLADRB1*:03:01 HLADRB1*:11:01 HLADRB1*:13:01	Neg	Specific
20	SEFLRFNRKLYSFDD	HLADRB1*:11:01 HLADRB1*:13:01 HLADRB1*:15:01	Neg	Specific
21	EFLRFNRKLYSFDDK	HLADRB1*:11:01 HLADRB1*:13:01	Neg	Specific
22	FLRFNRKLYSFDDKS	HLADRB1*:11:01 HLADRB1*:13:01	Neg	Specific
KNWEKYYEAVKKAMNIPNGFVWHEAVNWSPIKKQWVFLPRKCSDLPYNTETEENIGCNEIIIADAVFKTVKSIQIDKNPIDSASGFSSFKFLPDTNDQILIA				
23	KNWEKYYEAVKKAMN	HLADRB1*:11:01	Pos	Specific
24	WEKYYEAVKKAMNIP	HLADRB1*:04:01 HLADRB1*:07:01 HLADRB1*:11:01	Pos	Specific
25	EKYYEAVKKAMNIPN	HLADRB1*:11:01	Pos	Specific
26	KYYEAVKKAMNIPNG	HLADRB1*:11:01	Pos	Specific
27	NGFVWHEAVNWSPIK	HLADRB1*:04:01	Neg	Nonspecific
28	GFVWHEAVNWSPIKK	HLADRB1*:04:01 HLADRB1*:07:01	Neg	Nonspecific
29	AVNWSPIKKQWVFLP	HLADRB1*:07:01	Neg	Nonspecific
30	SPIKKQWVFLPRKCS	HLADRB1*:11:01	Pos	Nonspecific
31	PIKKQWVFLPRKCSD	HLADRB1*:11:01	Pos	Specific
32	IKKQWVFLPRKCSDL	HLADRB1*:11:01	Pos	Specific
33	KKQWVFLPRKCSDLP	HLADRB1*:11:01	Pos	Specific
34	KQWVFLPRKCSDLPY	HLADRB1*:11:01	Pos	Specific
35	QWVFLPRKCSDLPYN	HLADRB1*:11:01	Pos	Specific
36	IGCNEIIIADAVFKT	HLADRB1*:03:01	Neg	Specific
37	GCNEIIIADAVFKTV	HLADRB1*:03:01	Neg	Specific
38	CNEIIIADAVFKTVK	HLADRB1*:03:01	Neg	Specific
39	NEIIIADAVFKTVKS	HLADRB1*:03:01	Neg	Specific
40	EIIIADAVFKTVKSI	HLADRB1*:03:01	Neg	Specific
41	IIIADAVFKTVKSIQ	HLADRB1*:03:01	Neg	Nonspecific
42	IIADAVFKTVKSIQI	HLADRB1*:04:01 HLADRB1*:07:01 HLADRB1*:15:01	Neg	Nonspecific
43	IADAVFKTVKSIQID	HLADRB1*:04:01 HLADRB1*:07:01 HLADRB1*:15:01	Neg	Nonspecific
44	ADAVFKTVKSIQIDK	HLADRB1*:04:01 HLADRB1*:07:01 HLADRB1*:15:01	Neg	Nonspecific
45	DAVFKTVKSIQIDKN	HLADRB1*:04:01 HLADRB1*:07:01 HLADRB1*:15:01	Neg	Nonspecific
46	AVFKTVKSIQIDKNP	HLADRB1*:04:01 HLADRB1*:07:01 HLADRB1*:15:01	Neg	Nonspecific
47	VFKTVKSIQIDKNPI	HLADRB1*:07:01	Neg	Nonspecific
48	FKTVKSIQIDKNPID	HLADRB1*:04:01 HLADRB1*:07:01	Neg	Nonspecific
49	TVKSIQIDKNPIDSA	HLADRB1*:03:01	Pos	Nonspecific
50	VKSIQIDKNPIDSAS	HLADRB1*:03:01 HLADRB1*:04:01	Neg	Nonspecific
51	SFKFLPDTNDQILIA	HLADRB1*:03:01 HLADRB1*:04:01	Neg	Nonspecific
52	SSFKFLPDTNDQILI	HLADRB1*:04:01	Neg	Nonspecific
53	FSSFKFLPDTNDQIL	HLADRB1*:04:01	Neg	Nonspecific
NDKYEGIELLKNAKGLFKRYA				
54	NDKYEGIELLKNAKG	HLADRB1*:04:01	Neg	Specific
55	DKYEGIELLKNAKGL	HLADRB1*:11:01	Neg	Specific
56	KYEGIELLKNAKGLF	HLADRB1*:15:01	Neg	Specific
57	YEGIELLKNAKGLFK	HLADRB1*:04:01 HLADRB1*:11:01 HLADRB1*:15:01	Neg	Specific
58	EGIELLKNAKGLFKR	HLADRB1*:11:01	Neg	Specific
59	GIELLKNAKGLFKRY	HLADRB1*:03:01 HLADRB1*:04:01 HLADRB1*:11:01	Neg	Specific
60	IELLKNAKGLFKRYA	HLADRB1*:11:01 HLADRB1*:15:01	Neg	Specific
Miscellaneous				
61	KDGFKAEWATALDGK	HLADRB1*:04:01	Neg	Nonspecific

**Table 3:** HLA class II restricted specific and nonspecific epitopes derived from PsSP40 protein

No.	Peptide name	HLA specificity	IFN-γ induction	Specific/nonspecific
KSFNFAIIADLDKKSISKTDANNFKSIIKSGELTRV				
1	KSFNFAIIADLDKKS	HLADRB1*:03:01	Neg	Nonspecific
2	NFAIIADLDKKSISK	HLADRB1*:03:01 HLADRB1*:04:01	Neg	Nonspecific
3	FAIIADLDKKSISKT	HLADRB1*:11:01 HLADRB1*:03:01	Neg	Nonspecific
4	KKSISKTDANNFKSI	HLADRB1*:07:01	Pos	Specific
5	KTDANNFKSIIKSGE	HLADRB1*:15:01 HLADRB1*:11:01	Pos	Specific
6	DANNFKSIIKSGELT	HLADRB1*:11:01	Pos	Specific
7	ANNFKSIIKSGELTR	HLADRB1*:07:01	Neg	Specific
8	NNFKSIIKSGELTRV	HLADRB1*:03:01 HLADRB1*:07:01	Pos	Specific
KNWGVYYEAVKKAMNIPNGFVWHEAVNWSPIKKQWVFLPRKCSELPYNTETEENIGCNKIIIADAVFKTVKSIQIDKNPIDSASGFSSFKFLPDTNDQILIA				
9	KNWGVYYEAVKKAMN	HLADRB1*:11:01	Pos	Specific
10	NWGVYYEAVKKAMNI	HLADRB1*:11:01	Pos	Specific
11	WGVYYEAVKKAMNIP	HLADRB1*:04:01 HLADRB1*:07:01 HLADRB1*:11:01	Neg	Specific
12	GVYYEAVKKAMNIPN	HLADRB1*:04:01	Pos	Specific
13	VYYEAVKKAMNIPNG	HLADRB1*:11:01	Pos	Specific
14	NGFVWHEAVNWSPIK	HLADRB1*:04:01	Neg	Nonspecific
15	GFVWHEAVNWSPIKK	HLADRB1*:04:01 HLADRB1*:07:01	Neg	Nonspecific
16	AVNWSPIKKQWVFLP	HLADRB1*:07:01	Neg	Nonspecific
17	SPIKKQWVFLPRKCS	HLADRB1*:11:01	Pos	Nonspecific
18	PIKKQWVFLPRKCSE	HLADRB1*:11:01	Pos	Nonspecific
19	KKQWVFLPRKCSELP	HLADRB1*:11:01	Pos	Nonspecific
20	KQWVFLPRKCSELPY	HLADRB1*:11:01	Pos	Nonspecific
21	QWVFLPRKCSELPYN	HLADRB1*:11:01	Pos	Nonspecific
22	NKIIIADAVFKTVKS	HLADRB1*:03:01 HLADRB1*:04:01	Neg	Specific
23	IIADAVFKTVKSIQI	HLADRB1*:04:01 HLADRB1*:07:01 HLADRB1*:15:01	Neg	Nonspecific
24	DAVFKTVKSIQIDKN	HLADRB1*:04:01 HLADRB1*:07:01 HLADRB1*:15:01	Neg	Nonspecific
25	FKTVKSIQIDKNPID	HLADRB1*:04:01 HLADRB1*:07:01	Neg	Nonspecific
26	VKSIQIDKNPIDSAS	HLADRB1*:03:01 HLADRB1*:04:01	Neg	Nonspecific
27	SFKFLPDTNDQILIA	HLADRB1*:03:01	Neg	Nonspecific
28	SFNFAIIADLDKKSI	HLADRB1*:03:01 HLADRB1*:04:01	Neg	Nonspecific
29	AIIADLDKKSISKTD	HLADRB1*:03:01	Neg	Nonspecific
30	NGFVWHEAVNWSPIK	HLADRB1*:04:01	Neg	Nonspecific
31	IIIADAVFKTVKSIQ	HLADRB1*:03:01	Neg	Nonspecific
32	IADAVFKTVKSIQID	HLADRB1*:04:01 HLADRB1*:07:01 HLADRB1*:15:01	Neg	Nonspecific
33	ADAVFKTVKSIQIDK	HLADRB1*:04:01 HLADRB1*:07:01 HLADRB1*:15:01	Neg	Nonspecific
34	DAVFKTVKSIQIDKN	HLADRB1*:04:01 HLADRB1*:07:01 HLADRB1*:15:01	Neg	Nonspecific
35	AVFKTVKSIQIDKNP	HLADRB1*:04:01 HLADRB1*:07:01 HLADRB1*:15:01	Neg	Nonspecific
36	VFKTVKSIQIDKNPI	HLADRB1*:07:01	Neg	Nonspecific
37	FKTVKSIQIDKNPID	HLADRB1*:04:01 HLADRB1*:07:01	Neg	Nonspecific
38	TVKSIQIDKNPIDSA	HLADRB1*:03:01	Pos	Nonspecific
39	VKSIQIDKNPIDSAS	HLADRB1*:03:01 HLADRB1*:04:01	Neg	Nonspecific
40	SFKFLPDTNDQILIA	HLADRB1*:03:01 HLADRB1*:04:01	Neg	Nonspecific
41	SSFKFLPDTNDQILI	HLADRB1*:04:01	Neg	Nonspecific
42	FSSFKFLPDTNDQIL	HLADRB1*:04:01	Neg	Nonspecific
43	KDGFKAEWATAKDGK	HLADRB1*:04:01	POS	Nonspecific
44	PEKVIHTINTRDLTS	HLADRB1*:07:01	POS	Specific
45	LSEFLRFNCKLYSFD	HLADRB1*:13:01	Neg	Nonspecific

**Table 4:** HLA class II restricted specific and nonspecific epitopes derived from PsSP41 protein

No	Peptide name	HLA specificity	IFN-γ induction	Specific/nonspecific
GKSFKFAIIADLDKKSISKTDANNFKSIVKLGELTRV				
1	GKSFKFAIIADLDKK	HLADRB1*:04:01 HLADRB1*:07:01 HLADRB1*:15:01	Neg	Specific
2	KSFKFAIIADLDKKS	HLADRB1*:03:01	Neg	Specific
3	SFKFAIIADLDKKSI	HLADRB1*:03:01 HLADRB1*:04:01	Neg	Specific
4	KFAIIADLDKKSISK	HLADRB1*:03:01 HLADRB1*:04:01	Neg	Specific
5	FAIIADLDKKSISKT	HLADRB1*:03:01 HLADRB1*:11:01	Neg	Nonspecific
6	TDANNFKSIVKLGEL	HLADRB1*:13:01	Neg	Nonspecific
7	DANNFKSIVKLGELT	HLADRB1*:13:01	Neg	Nonspecific
8	ANNFKSIVKLGELTR	HLADRB1*:11:01	Neg	Nonspecific
9	NNFKSIVKLGELTRV	HLADRB1*:13:01	Pos	Specific
10	YEAVKKAMNMPNGFV	HLADRB1*:04:01	Neg	Specific
WEKYYEAVKKAMNMPNGFVWHEAVNWSPIKKQWVFLPRKCSELPYNTETEENIGCNEIIIADERFNTVKSIQIGRIPIDSASGFSSFKFLPDTNDEILIALKTVERMINCYY				
11	WEKYYEAVKKAMNMP	HLADRB1*:07:01 HLADRB1*:11:01	Pos	Specific
12	EKYYEAVKKAMNMPN	HLADRB1*:11:01	Pos	Specific
13	GFVWHEAVNWSPIKK	HLADRB1*:04:01 HLADRB1*:07:01	Neg	Nonspecific
14	KKQWVFLPRKCSELP	HLADRB1*:11:01	Pos	Nonspecific
15	GCNEIIIADERFNTV	HLADRB1*:03:01	Neg	Specific
16	CNEIIIADERFNTVK	HLADRB1*:03:01	Neg	Specific
17	NEIIIADERFNTVKS	HLADRB1*:03:01	Neg	Specific
18	EIIIADERFNTVKSI	HLADRB1*:03:01	Neg	Specific
19	IIIADERFNTVKSIQ	HLADRB1*:03:01	Neg	Specific
20	IIADERFNTVKSIQI	HLADRB1*:04:01 HLADRB1*:07:01	Neg	Specific
21	DERFNTVKSIQIGRI	HLADRB1*:04:01 HLADRB1*:07:01	Neg	Specific
22	ERFNTVKSIQIGRIP	HLADRB1*:04:01 HLADRB1*:07:01	Neg	Specific
23	RFNTVKSIQIGRIPI	HLADRB1*:07:01	Neg	Specific
24	FNTVKSIQIGRIPID	HLADRB1*:07:01	Neg	Specific
25	SFKFLPDTNDEILIA	HLADRB1*:03:01	Neg	Specific
26	LIALKTVERMINCYY	HLADRB1*:11:01	Pos	Specific
27	KDGFKAEWATAKDGK	HLADRB1*:04:01	POS	Nonspecific
28	KAEWATAKDGKMYVG	HLADRB1*:04:01	Neg	Nonspecific
29	SEFLRFNCKLYSFDD	HLADRB1*:11:01	Neg	Nonspecific
Miscellaneous				
30	GAELSEFLRFNCKLY	HLADRB1*:15:01	Neg	Nonspecific

**Table 5:** HLA class II restricted specific and nonspecific epitopes derived from PsSP40, PsSP41, and PsSP42 protein in mouse

Peptide name	HLA specificity	IFN-γ induction	Specific/nonspecific
PsSP40			
NWGVYYEAVKKAMNI	H2-IE-d	Pos	Specific
KNWGVYYEAVKKAMN	H2-IE-d	Pos	Specific
WGVYYEAVKKAMNIP	H2-IE-d	Neg	Specific
NKNWGVYYEAVKKAM	H2-IE-d	Neg	Specific
GVYYEAVKKAMNIPN	H2-IE-d	Pos	Specific
KVLWPEKVIHTINTR	H2-IE-d	Pos	Specific
PsSP41			
WEKYYEAVKKAMNMP	H2-IE-d	Pos	Specific
EKYYEAVKKAMNMPN	H2-IE-d	Pos	Specific
PsSP42			
NWEKYYEAVKKAMNI	H2-IE-d	Pos	Specific
WEKYYEAVKKAMNIP	H2-IE-d	Pos	Specific
EFLRFNRKLYSFDDK	H2-IE-d	Neg	Specific
ELSEFLRFNRKLYSF	H2-IE-d	Neg	Specific
EKYYEAVKKAMNIPN	H2-IE-d	Pos	Specific
IKKQWVFLPRKCSDL	H2-IE-d	Pos	Specific
KKQWVFLPRKCSDLP	H2-IE-d	Pos	Specific
PIKKQWVFLPRKCSD	H2-IE-d	Pos	Specific

### Preparation of the recombinant *L. tarentolae* expressing PsSP42

**Fig. 2:**
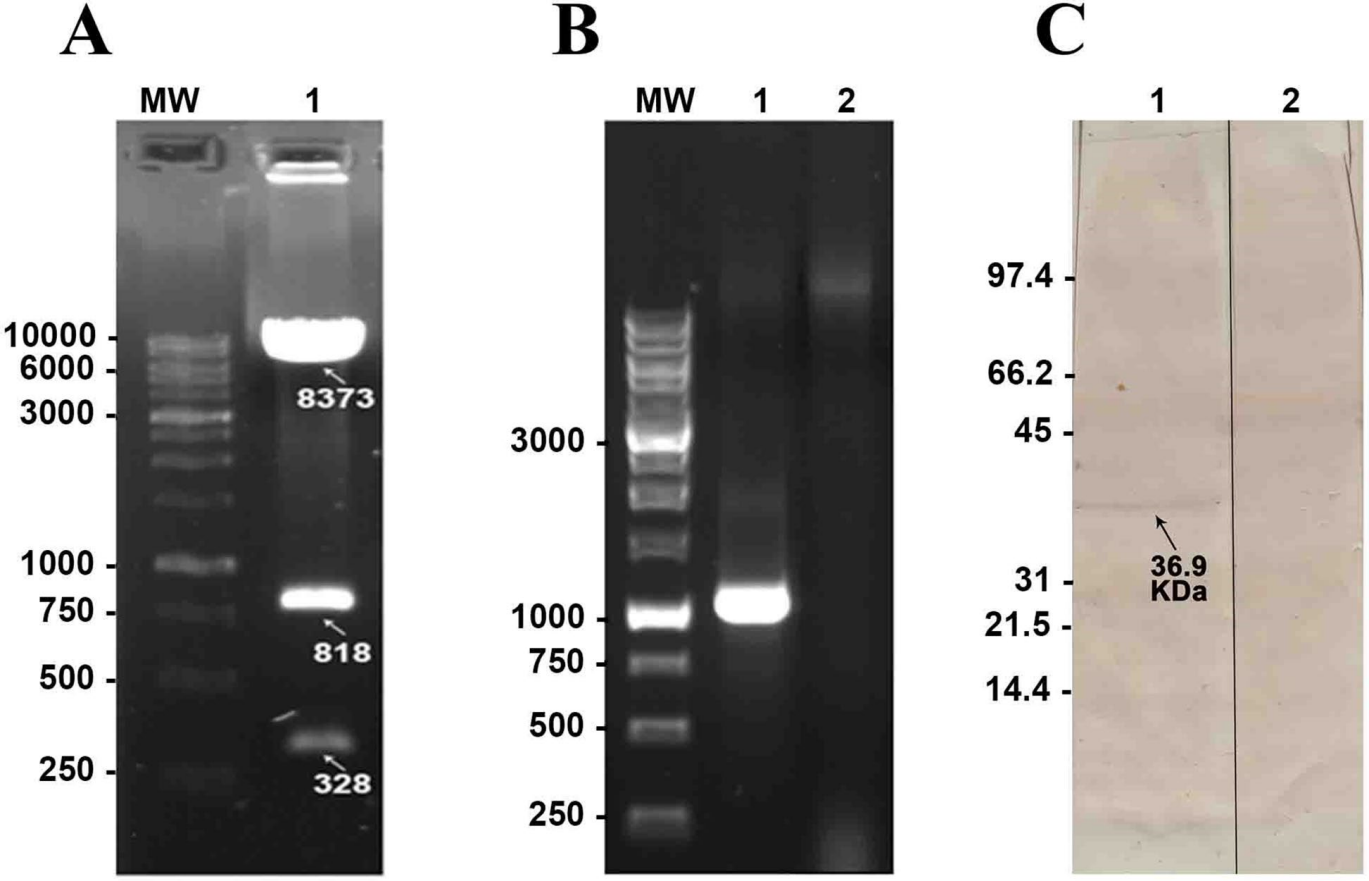
Generation of *L. tarentolae* expressing PsSP42. Recombinant *L. tarentolae* transiently expressing PsSP42 was generated through episomal pLEXSY-PsSP42 transfection. **A** Confirmation of pLEXY-PsSP42 construct. Enzymatic digestion of pLEXY-PsSP42 was done with *Bgl*II, and three separated bands were achieved (lane 1). **B** Confirmation of the presence of PsSP42 gene in the recombinant *L. tarentolae*-PsSP42 by PCR. Amplification of the 1100-bp fragment (lane 1). Non-recombinant *L. tarentolae* was used as negative control (lane 2). **C** Confirmation of PsSP42 protein expression in recombinant *L. tarentolae*-PsSP42 using Western blot analysis. A 39.6-kDa band corresponding to the PsSP42 protein was detected in the cell pellet of recombinant *L. tarentolae*-PsSP42 using anti-His antibody (lane 1), and lane 2 is the non-recombinant *L. tarentolae* as negative control. MW, molecular weight

To prepare the recombinant *L. tarentolae*, the PsSP42 fragment was primarily subcloned in the *Sal*I and *Not*I sites of the pLEXSY-neo2 vector. Enzymatic digestion of pLEXY-PsSP42 at the *Bgl*II restriction site confirmed successful recombination with three separate bands (Fig. 2A, lane 1). Recombinant *L. tarentolae* transiently expressing PsSP42 was generated through episomal pLEXSY-PsSP42 transfection (as described in “Materials and Methods” secton). Presence of the PsSP42 gene sequence in recombinant episomal *L. tarentolae*-PsSP42 was assayed using PsSP42-specific primers in PCR reaction. Amplification of the 1100-bp fragment for PsSP42 gene in *L. tarentolae*-PsSP42 was confirmed (Fig. 2B, lane 1); non-recombinant *L. tarentolae* was included as negative control (Fig. 2B, lane 2). The expression of PsSP42 protein (a hydrophilic protein of 39.6 kDa weight and PI of 8. 94) in the cell pellet of recombinant *L. tarentolae*-PsSP42 was confirmed using anti-His-tag antibody by Western blot analysis (Fig. 2C, lane 1). Non-recombinant *L. tarentolae* was included as negative control (Fig. 2C, lane 2).

### Pre-infectious challenge cytokine profile determination

Five groups of BALB/c mice (G1-G5) were immunized twice at 3-week intervals with VR1020-PsSP42, NTC-PsSP42, VR1020, NTC, or PBS, respectively, in hind footpad. Three weeks thereafter, all animals were subcutaneously challenged with 2 × 10^7^ late stationary phase of *L*. *tropica* along with 0.5 pairs of *Ph*. *sergenti* SGH in the contralateral footpad (Table 1).

**Fig. 3:**
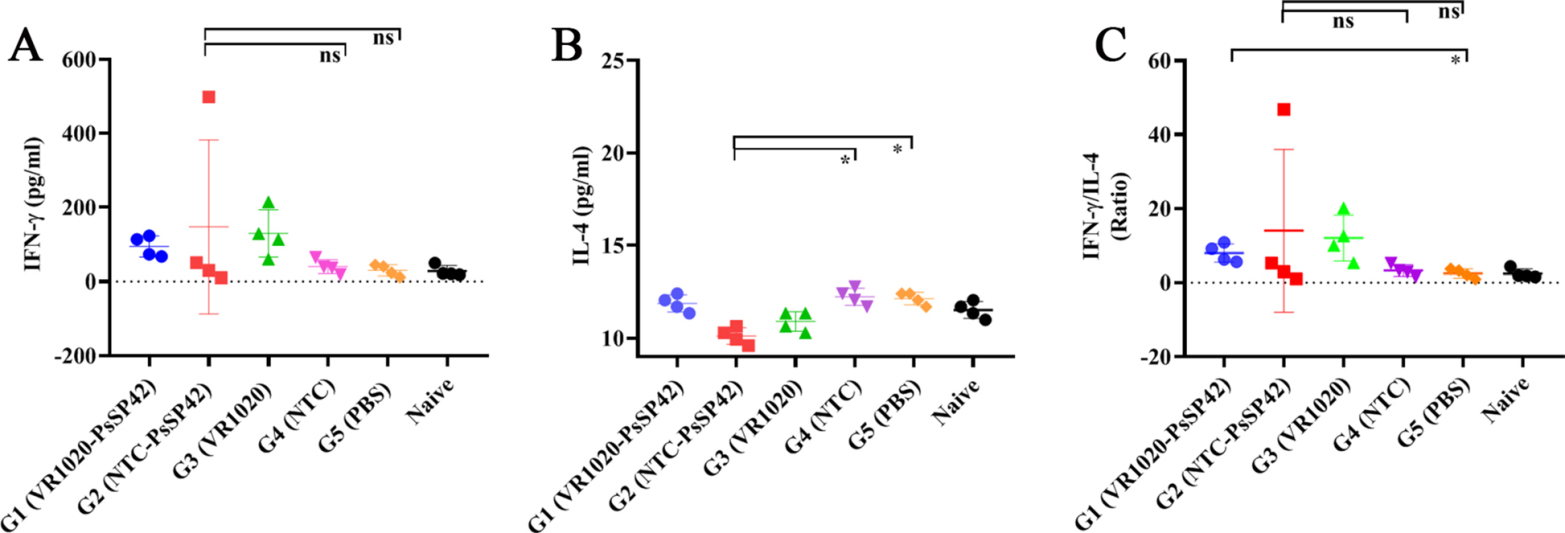
Before-challenge analysis of cytokine production in immunized and control BALB/c mice. Analysis of cytokines production in vaccinated, control, and naive mice after the last immunization and prior to infection with *L. tropica* + SGH using ELISA. Four mice per group were sacrificed and splenocytes were restimulated with *L. tarentolae*-PsSP42 F/T antigen. **A** IFN-γ production, **B** IL-4 production, **C** IFN-γ/IL-4 ratio. The results are presented as mean ± SD (in pg/ml), and Student’s *t*-test was used for statistical analysis. (*P < 0.05; ns, nonsignificant)

After the last immunization and prior to *L. tropica* + SGH challenge, four mice in each group were euthanized and the spleens were dissected. The splenocytes were in vitro restimulated with F/T antigen of recombinant* L. tarentolae-*PsSP42, and two cytokines including IFN-γ and IL-4 were measured in the culture supernatants by ELISA (Fig. 3 and Additional file 6, Table S3). As shown in Fig. 3A, IFN-γ production by G1 group was significantly higher than PBS but not the relevant G3 group. The IFN-γ production in G2 group was higher compared with G4 (the relevant control) and PBS, but not significantly. Therefore, the pre-challenge IFN-γ secretion was not informative. As indicated in Fig. 3B, the IL-4 induction by NTC-PsSP42 immunizations was lowered in G2 group compared with the relevant controls (G4 and G5). Instead, G1 failed to downmodulate this cytokine compared with the relevant controls (G3 and G5). As a result, on the basis of the pre-challenge data, we could not define a remarkable skewness toward Th1 response by any of the plasmid constructs (the Th1/Th2 ratio indicated in Fig. 3C). The reason could be attributed to the intrinsic characteristics of DNA immunizations, which typically result in a low level of cytokine, barely detectable through ELISA assay before challenge (as repeatedly indicated in our previous experiments) [43, 44].

### Post-infectious challenge cytokine profile determination

**Fig. 4:**
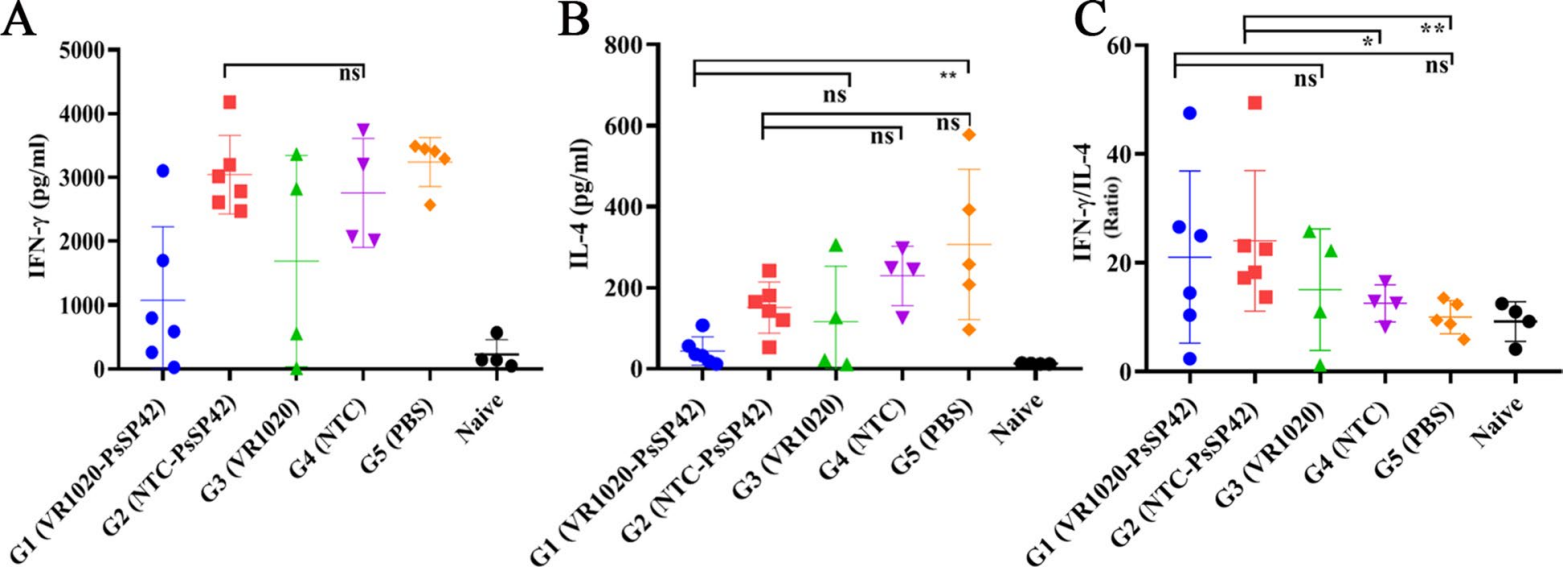
After-challenge analysis of cytokine production in immunized and control BALB/c mice. Analysis of cytokines production in vaccinated, control, and naive mice at 8 weeks post-infection with *L. tropica* + SGH using ELISA. Four to six mice per group were sacrificed and splenocytes were restimulated with *L. tropica* F/T antigen. **A** IFN-γ production, **B** IL-4 production, **C** IFN-γ/IL-4 ratio. The results are presented as mean ± SD (in pg/ml), and Student’s *t*-test was used for statistical analysis. (*P < 0.05, **P < 0.01; ns, nonsignificant)

To determine the protective effect of DNA vaccine constructs against *L. tropica* + SGH infection, IFN-γ and IL-4 were also measured 8 weeks post-challenge. Four to six mice per group were ethically euthanized and the splenocytes were restimulated with *L. tropica* F/T antigen. IFN-γ and IL-4 were measured in the culture supernatants (5 and 3 days post culture, respectively) by ELISA (Fig. 4 and Additional file 7: Table S4). As indicated in Fig. 4A, the IFN-γ production was lower in G1 group compared with pertinent controls (G3 and G5). Meanwhile, the IFN-γ production in G2 group was not significantly higher compared with pertinent G4 group.

Meanwhile, the highest level of IL-4 was detected in PBS group, as expected. Both G1 and G2 were able to reduce the level of IL-4 production (although not statistically significantly) compared with their empty vectors (Fig. 4B). As indicated in Fig. 4C, the IFN-γ to IL-4 ratio of NTC-PsSP42 immunized mice showed a higher level compared with its relevant controls (G2 and G5). On the other hand, the IFN-γ to IL-4 ratio after VR1020-PsSP42 immunization was non-significantly higher compared with relevant control groups (G3 and G5).

### Parasite load determination after infectious challenge

**Fig. 5:**
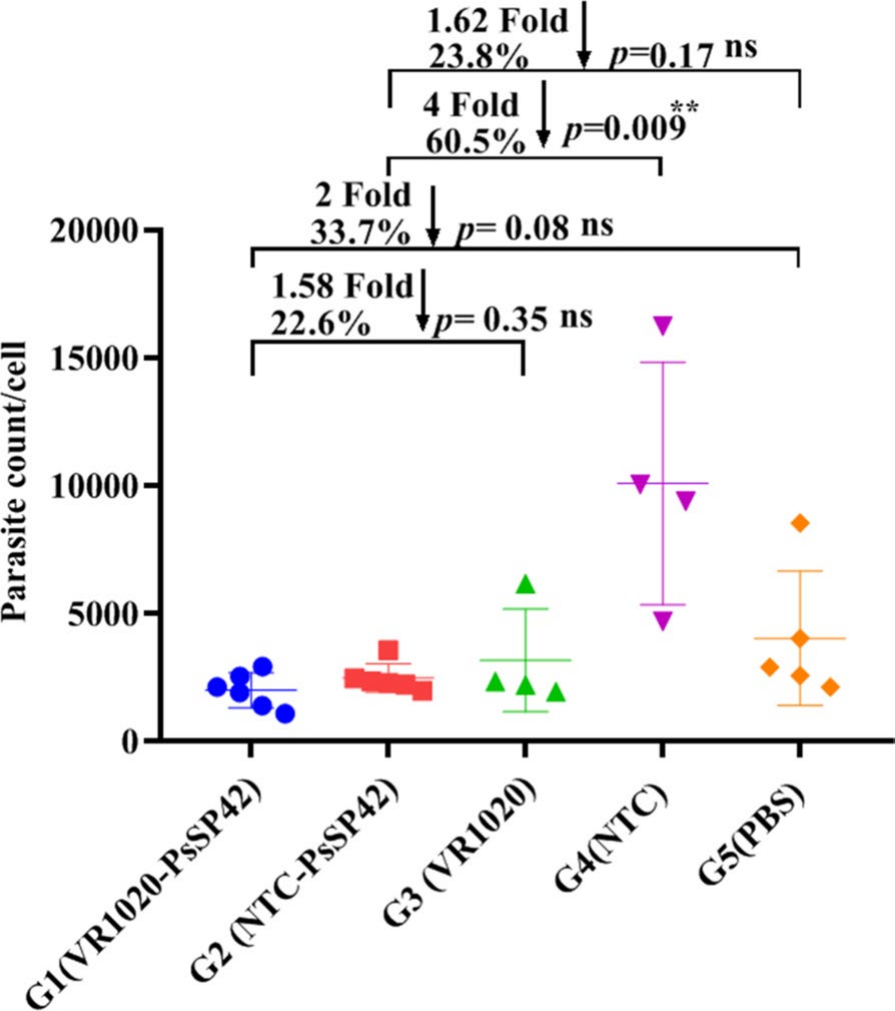
Parasite load quantification by qRT-PCR. BALB/c mice were immunized in the left footpad two times at 3-week intervals with two different DNA plasmids encoding for *Ph. sergenti* apyrase salivary PsSP42 (VR1020-PsSP42 and NTC-PsSP42), empty plasmids (VR1020 and NTC), or PBS (control). All animals were subcutaneously challenged with 2 × 10^7^ late stationary phase of *L. tropica* along with 0.5 pairs of *Ph. sergenti* SGH in the right footpad. Eight weeks post challenge, the number of parasites per cell was determined by qRT-PCR from each individual lymph node (*n* = 4–6 mice per group). Student’s *t*-test was used for statistical analysis. (*P < 0.05, **P < 0.01; ns, nonsignificant). All data are presented as mean ± SD. The data presented here are representative of two independent experiments. (The parasite burden dataset of the second experiment is given in Additional File 9, Fig. S4)

The potency of NTC-PsSP42 and VR1020-PsSP42 was estimated by quantification of parasite burden at 8 weeks post challenge using real-time PCR method (Fig. 5 and Additional file 8, Table S5). As shown, the parasite load was significantly lower in NTC-PsSP42 immunized group compared with NTC control group. In other words, NTC-PsSP42 immunization made a fourfold or 60% reduction in parasite load compared with NTC control. The parasite load in NTC-PsSP42 immunized mice was reduced by 23% (1.62 fold) compared with the PBS control group, but this difference was not significant. On the other hand, the parasite load reduction by VR1020-PsSP42 immunization was not significant compared with the pertinent control groups (22.6% and 33.7% compared with VR1020 and PBS controls, respectively). Therefore, contrary to VR1020-PsSP42, NTC-PsSP42 immunization relatively reduced the parasite burden in the lymph node of *L. tropica* infected BALB/c mice. It was further speculated that the partial parasite load control by NTC-PsSP42 immunization after *L. tropica* infection could correlate with a weak Th1 skewness in this group compared with VR1020-PsSP42 immunized group.

## Discussion

It has been proven for decades that Th1 cellular immune responses and IFN-γ secretion are the key players in controlling *Leishmania* parasite infection [15]. At present, considerable progress has been made to identify potential candidate antigens for developing vaccines against leishmaniasis [46]. Nevertheless, no licensed human vaccine is yet available. One major explanation is our limited understanding of the complex interaction between the host, pathogen, and vector, and particularly the role of the sand fly-related factors [47]. In recent years, some sand fly salivary deposited components through sand fly proboscis have been revealed to induce Th1 responses [13] and therefore have moved to the top of the vaccine candidate list as targets for a prophylactic vaccine. In some rodent experimental models, immunization with salivary gland homogenate or salivary components, as well as pre-exposure to uninfected sand fly bite, elicited Th1 responses and protected against *Leishmania* species [13, 39]. Oliveira et al., in 2015, indicated that exposure of nonhuman primate (NHP) to uninfected sand fly bites or immunizing with a defined recombinant salivary protein, rPdSP15, controlled sand fly-transmitted *L. major* infection compared with controls, suggesting that immunity to saliva or PdSP15 augments the host immune response to the parasites while maintaining minimal pathology [48]. These findings suggested that the salivary components of sand fly may play a critical role in the host–parasite interaction and have implications for novel vaccine candidates [35, 49–51].

Given that the salivary gland proteins derived from *Ph. sergenti* have been less investigated as vaccine candidates, Gholami et al. screened the immunogenicity of 14 different proteins including apyrase family members in DNA platform [45]. The study indicated that epidermal pre-immunization with recombinant VR1020 expressing PsSP40, PsSP41, and PsSP42 (without electroporation) and then exposure to *Ph. sergenti* derived SGH resulted in comparable IFN-γ and IL-4 induction and IFN-γ/IL-4 ratio without preference among the three. On the basis of these results, in our recent study, we attempted to evaluate the protective potential of apyrase from *Ph. sergenti*, as vaccine candidate. We analyzed multiple characters of the proteins and found no specific difference in three-dimensional structure or overall antigenicity, physicochemical characters, or virtual immune response profile, despite the subtle difference in primary sequence of the three proteins. Therefore, to select among the proteins as a vaccine candidate for further in vivo study, the HLA class II peptide content with IFN-γ production ability was measured. The epitope content for comparing candidates together and selecting among them was already introduced by other studies as “fishing for antigens using epitopes as bait.” To select antigens for vaccine design, it is important to consider the overall potential for immunogenicity, which is directly related to cytotoxic T cell or T helper cell epitope content. In other words, the greater the T cell epitope content of an antigen, the more likely it will induce an immune response [42, 52]. Therefore, on the basis of in silico HLA class II-binding peptide analysis, PsSP42 was distinguished with more specific epitopes with IFN-γ inducing potential and was selected among the three family members for further evaluation as a vaccine candidate in BALB/c model.

To this end, the efficacy of conventional (antibiotic gene dependent) VR1020 plasmid along with nanoplasmid as a new-generation plasmid devoid of antibiotic resistance gene, both expressing PsSP42 antigen as target candidate, was evaluated in BALB/c mice. Both plasmids are CMV-promoter dependent for protein production. However, NTC9385R has more advantages over VR1020, such as smaller molecular size (1.7 versus 5.7 bp) and chimeric promoter for enhancing protein production.

The cellular immune response was measured by measuring the splenocyte activation before and after infectious challenge along with SGH. Although we could barely measure a Th1 deviation before challenge, NTC-PsSP42 immunization resulted in significant IFN-γ to IL-4 ratio compared with pertinent controls in contrast to VR1020-PsSP42 after challenge. This could have been the underlying reason for a relative parasite load reduction following NTC-PsSP42 immunization.

The results of Nandadeva Lokugamage et al. showed the effectiveness of nanoplasmid encoding *Tripanosoma cruzi TcG2/TcG4* antigens (Tc.nano2/4) by increasing the function of CD4^+^ and CD8^+^ cells that express IFN-γ and perforin, leading to the control of chronic parasite persistence [53]. In another experimental study, this group indicated that nanoplasmid encoding same antigens was more effective for early activation and production of IFN-γ by CD4^+^ effector/effector memory cells and also *nano2/4* C57BL/6 immunized mice exhibited potent control of parasite burden compared with conventional pcDNA3.1 [54]. The lack of potent Th1 response in our experiment after NTC-PsSP42 immunization could be linked to the NTC structure. The NTC plasmid has a shorter prokaryotic region compared with conventional plasmids, which could reduce the number of CpG motifs mainly found on prokaryotic sequences. The addition of immunostimulatory sequences, such as CpG oligonucleotide, to the nanoplasmid immunization may serve to improve its efficacy. As evidenced by Suschak et al., the Venezuelan equine encephalitis virus (VEEV) and Ebola virus (EBOV) glycoproteins were investigated as a DNA vaccine in two distinct platforms, namely nanoplasmid and conventional pWRG7077. Their results indicated that the utilization of nanoplasmid vectors could enhance the immunogenicity and protective efficacy of both alpha virus and filovirus DNA vaccines when complemented with immunostimulatory sequences [55].

Although NTC-PsSP42 weakly controlled parasite load, we support this platform mainly because of the minimal size of the plasmid. Both NTC-PsSP42 (2774 bp) and VR1020-PsSP42 (6036 bp) constructs were transferred through identical electroporation approach, so it can be inferred that the smaller size of NTC-PsSP42 enhances the delivery into target cells. Of note, we have compared the expression level of NTC-PsSP42 protein delivered by in vitro electroporation into COS-7 cells versus VR1020-PsSP42 expression. The outcome confirmed the comparable expression level between the two constructs [36]. However, taking advantage of the in vivo electroporation, the efficiency is technically more complicated and requires pre-optimization to reach the optimum pulse length and strength, number of pulses, and the intervals for each individual plasmid. Obviously, the electroporation efficiency can more strongly impact the immunization results of large plasmids than small ones such as nanoplasmids [56–58]. This is more important, especially when a subcutaneous route is used for vaccine delivery instead of epidermal route, where antigen-presenting cells predominate.

In this study, we attributed the suboptimal immunity associated with each immunization protocol to an insufficiently elevated Th1 versus Th2 response, which is marked by the well-characterized IL-4 cytokine. This conclusion is based on a longstanding assumption that early and sustained production of IL-4 in *L. major*-infected BALB/c mice hinders Th1 differentiation and predisposes these mice to nonhealing infection [59]. However, the role of IL-4 and IL-4Rα in mouse models of *Leishmania* infection is controversial. IL-4 is no longer regarded as the sole mediator of the Th2 response; rather, the combined effects of IL-4 and IL-13 increase susceptibility to *L. major* [60]. Both cytokines share a common IL-4Ra chain, which is expressed on various innate and adaptive immune cells [61]. Importantly, both cytokines have also been highlighted for their immunoregulatory functions and potential to promote Th1 responses in infections caused by different *Leishmania* species [62]. A comparison of *L. tropica* and *L. major* infections indicates that the patterns of immune response are species-specific, with varying effects based on host susceptibility genes [63]. This indicates that distinct families of cytokines serve specific functions in mediating the immune response against various species of infectious parasites, as well as in response to specific antigens incorporated in vaccine formulations. The implications of these findings are substantial for the development of vaccines aimed at targeting different species of *Leishmania*. To optimize vaccine efficacy, it is essential to obtain a more detailed and comprehensive cytokine profile following immunization, as well as during the early stages after pathogen challenge. This approach will significantly enhance the translation of research findings into clinical practice, ultimately leading to more effective treatment and prevention strategies against leishmaniasis.

There is ample evidence showing the immunogenicity of apyrase protein as vaccine candidate [21]. Taken together, we believe that *Ph. sergenti*-derived PsSP42 has potential as a vaccine candidate if used in a suitable platform. The NTC-PsSP42 formulated with immunostimulatory oligonucleotides as CpG can act as an alternative to compensate the low immunogenicity of the construct. We can even use heterologous prime-boost regimens to improve the potential of viral delivery systems in booster dose. It is important to note once again that new-generation plasmids with antibiotic resistance gene-free backbones are worth evaluation and substitution for conventional plasmids because of the regulatory hindrances that inhibit human use of DNA vaccines. DNA vaccines offer a promising advancement in immunization technology by prolonging the presentation of antigens within living organisms. This extended antigen exposure makes them a potential alternative to conventional vaccines that utilize whole pathogens. Despite this exciting potential, significant gaps in our understanding remain, particularly regarding the new generation of plasmids used in these vaccines. In this report, we detail the findings from an exploratory study focused on vaccination against *L. tropica*, and it is necessary to undertake further investigations to deepen our insights and optimize vaccine efficacy.

## Supplementary Information

Additional file 1: Fig S1. Multiple sequence alignment of PsSP40, PsSP41, and PsSP42. The stars indicate identical amino acids.

Additional file 2: Fig S2: The 3D structure of apyrase proteins predicted by I-TASSER server. The C-score is a measure of prediction confidence.

Additional file 3: Table S1: Physicochemical parameters of PsSP40, PsSP41, and PsSP42.

Additional file 4: Table S2: Allergenicity and antigenicity evaluation of PsSP40, PsSP41, and PsSP42.

Additional file 5: Fig S3: The outputs of immune simulation using the C-ImmSim server for apyrase stimulation. The immunogenicity of the three apyrase proteins was evaluated using the C-ImmSim server after two injections at time steps 1 and 63 with 3-week intervals. Column A indicates cytokine responses. The level of cytokines is illustrated in the main plot. In addition, the inset plot (upper right) indicates the IL-2 level and the diversity index (D). Column B indicates T helper cell populations. The resting state implies cells not presented with the antigen, while duplicating state indicates cells in the mitotic cycle. The anergic state shows the T-cell tolerance to the antigen (a, c, e); differentiated T cell clones are indicated in b, d, and f plots.

Additional file 6: Table S3: Mean ± SD of cytokine production and raw data for each immunized and control groups before *L. tropica* + SGH challenge.

Additional file 7: Table S4: Mean ± SD of cytokine production and raw data for each immunized and control groups after challenge with *L. tropica* + SGH.

Additional file 8: Table S5: Mean ± SD of Parasite burden and raw data for each immunized and control group after challenge with *L. tropica* + SGH.

Additional file 9: Fig S4: Parasite load quantification by qRT-PCR. (Second experiment). BALB/c mice were immunized in the left footpad two times at 3-week intervals with two different DNA plasmids encoding for *Ph. sergenti* apyrase salivary PsSP42 (VR1020-PsSP42 and NTC-PsSP42), empty plasmids (VR1020 and NTC), or PBS (control). All animals were subcutaneously challenged with 2 × 10^7^ late stationary phase of *L. tropica* along with 0.5 pairs of *Ph. sergenti* SGH in the right footpad. Eight weeks post challenge, the number of parasites per cell was determined by qRT-PCR from each individual lymph node (six mice per group). Student’s *t*-test was used for statistical analysis. (* *p*<0.05, ** *p*<0.01; ns, nonsignificant). All data are presented as mean ± SD. The data presented here are representative of two independent experiments. (The parasite burden dataset of the second experiment is given in Additional File 9, Fig. S4).

## Data Availability

No datasets were generated or analyzed during the current study.
