# Supplementary figures and images for: Evaluating the effect of immunization with DNA encoding Phlebotomus sergenti apyrase protein (PsSP42) against Leishmania tropica infection in BALB/c mouse model
Source: Parasit Vectors. 2026 Mar 9;19:163. doi: 10.1186/s13071-026-07255-x (PMC13085537; doi:10.1186/s13071-026-07255-x)

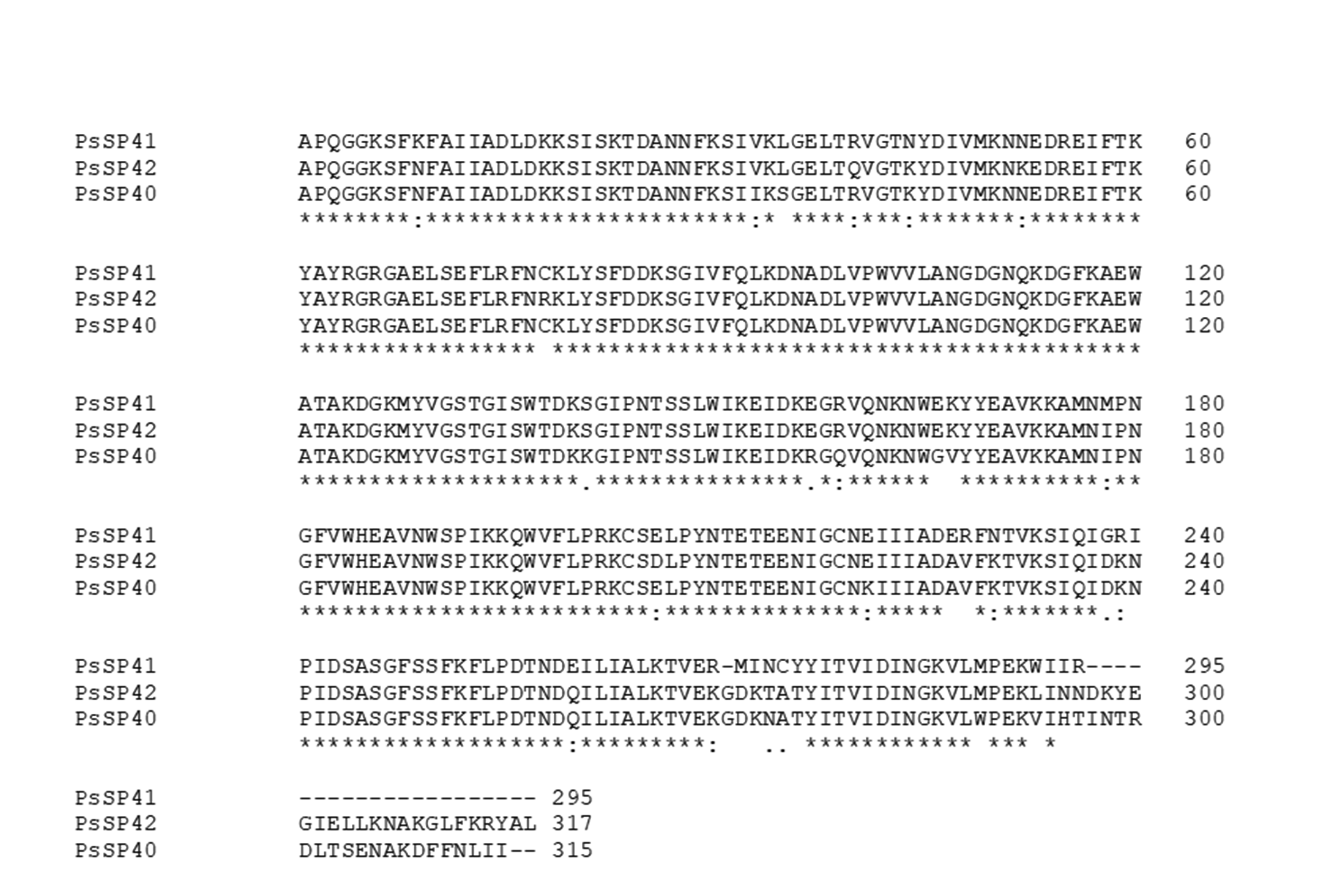

Supplement: Supplementary file 1 — Additional file 1: Fig S1. Multiple sequence alignment of PsSP40, PsSP41, and PsSP42. The stars indicate identical amino acids. [file 13071_2026_7255_MOESM1_ESM.tif]

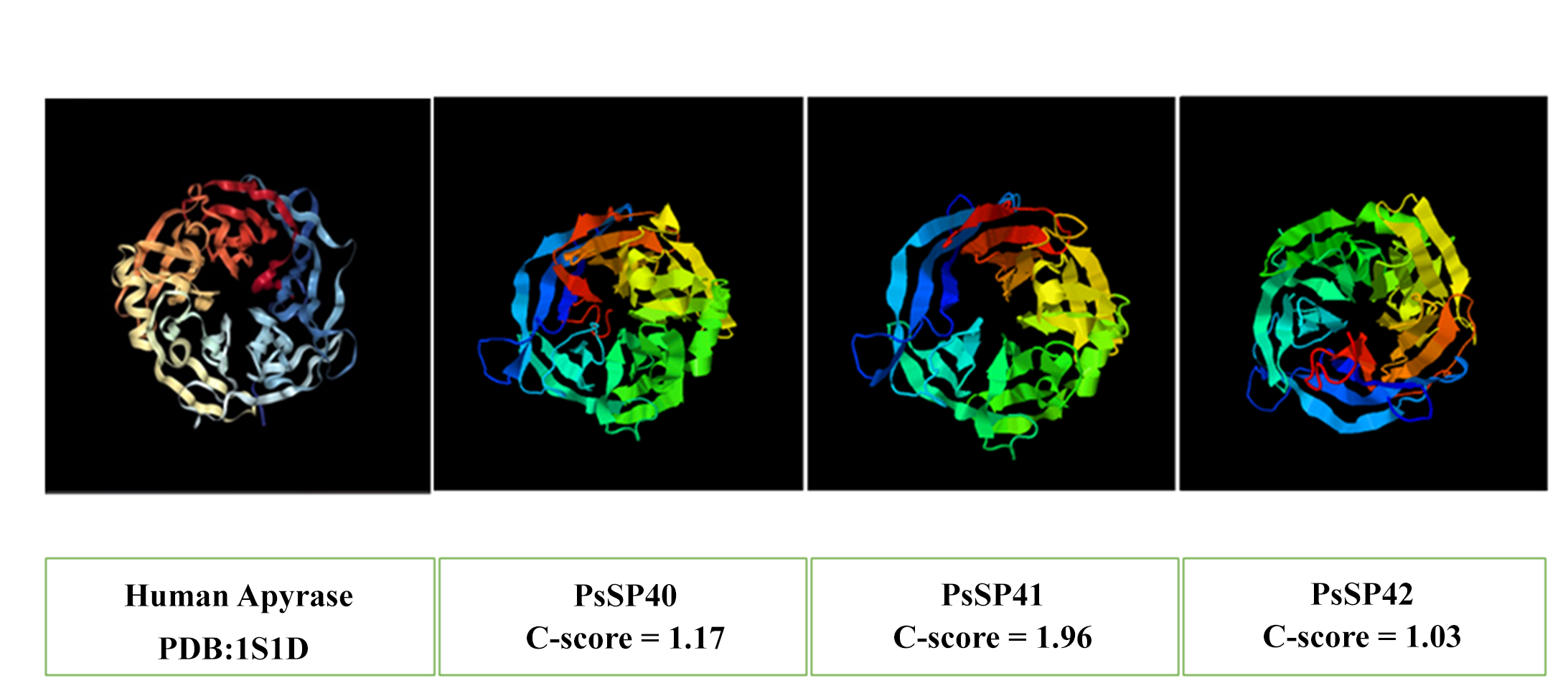

Supplement: Supplementary file 2 — Additional file 2: Fig S2: The 3D structure of apyrase proteins predicted by I-TASSER server. The C-score is a measure of prediction confidence. [file 13071_2026_7255_MOESM2_ESM.tif]

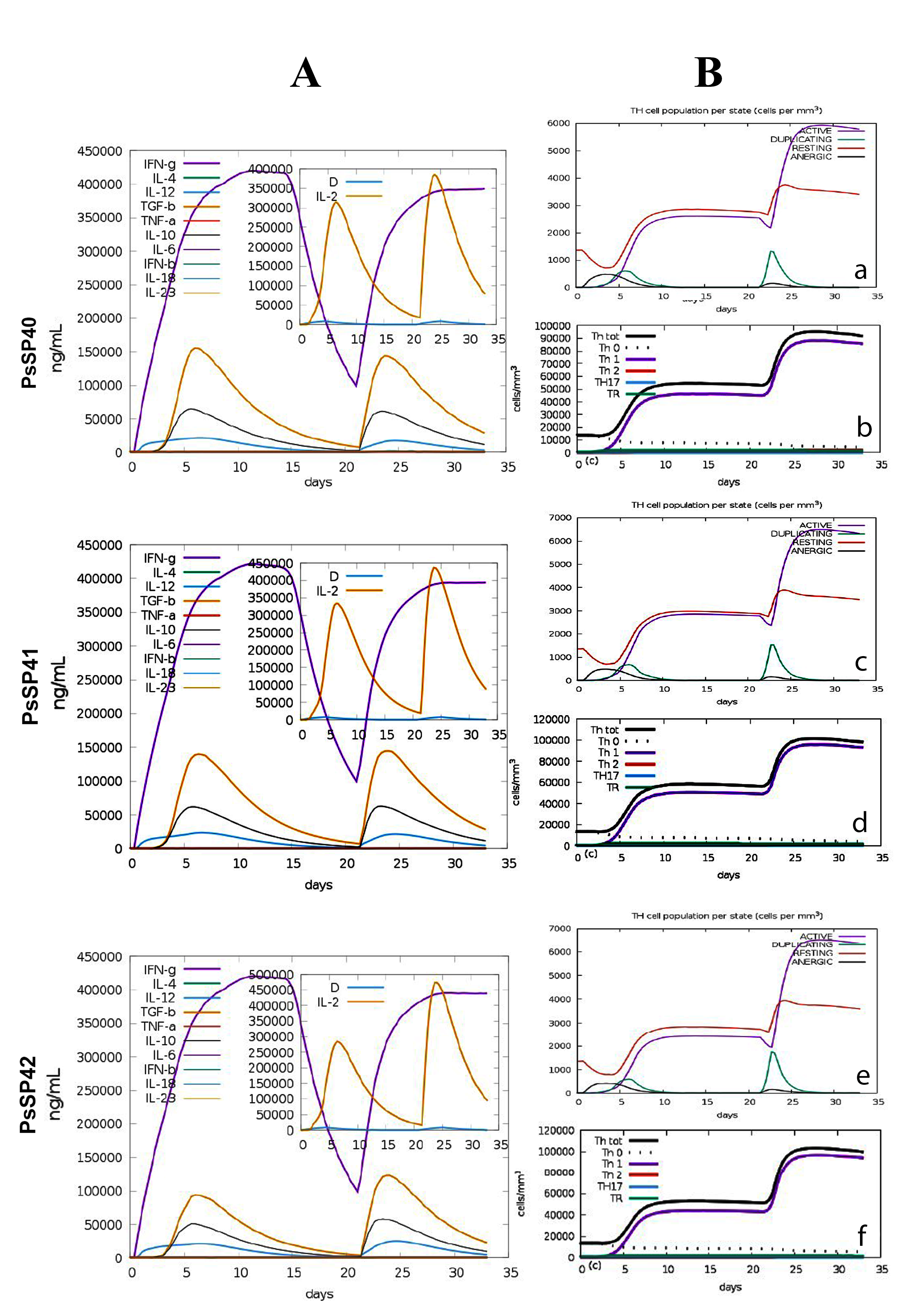

Supplement: Supplementary file 5 — Additional file 5: Fig S3: The outputs of immune simulation using the C-ImmSim server for apyrase stimulation. The immunogenicity of the three apyrase proteins was evaluated using the C-ImmSim server after two injections at time steps 1 and 63 with 3-week intervals. Column A indicates cytokine responses. The level of cytokines is illustrated in the main plot. In addition, the inset plot (upper right) indicates the IL-2 level and the diversity index (D). Column B indicates T helper cell populations. The resting state implies cells not presented with the antigen, while duplicating state indicates cells in the mitotic cycle. The anergic state shows the T-cell tolerance to the antigen (a, c, e); differentiated T cell clones are indicated in b, d, and f plots. [file 13071_2026_7255_MOESM5_ESM.tif]

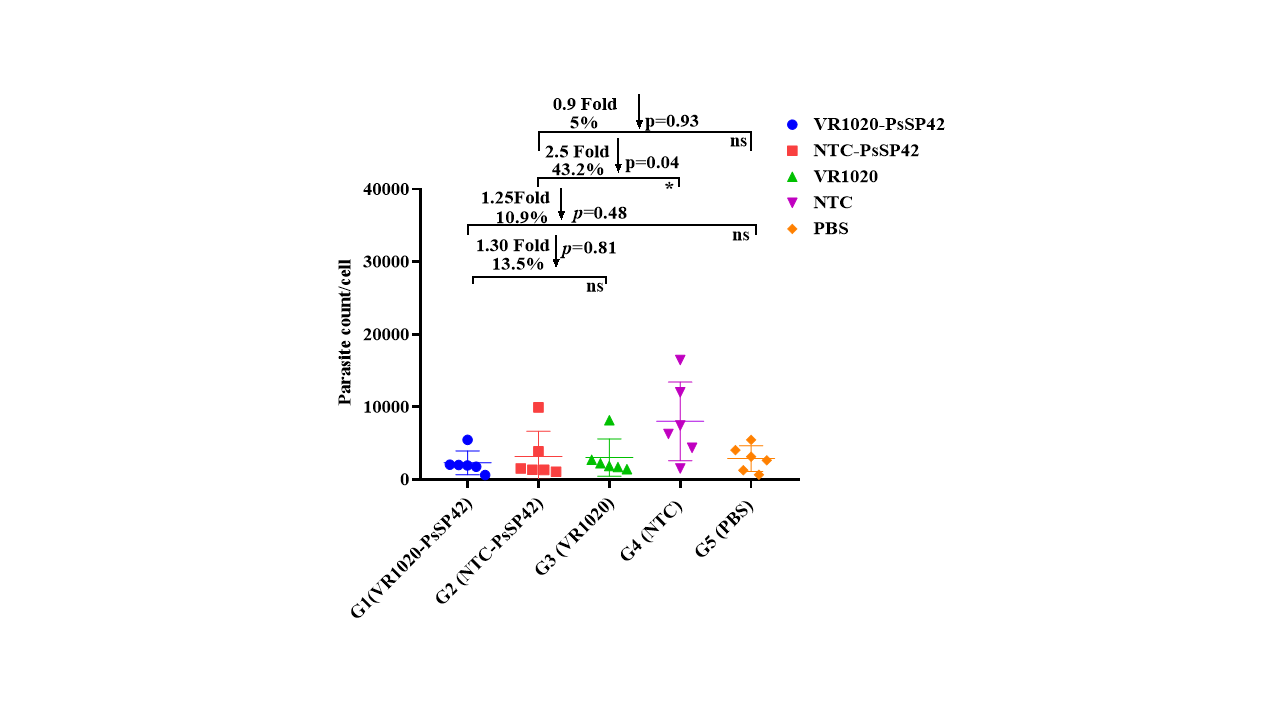

Supplement: Supplementary file 9 — Additional file 9: Fig S4: Parasite load quantification by qRT-PCR. (Second experiment). BALB/c mice were immunized in the left footpad two times at 3-week intervals with two different DNA plasmids encoding for Ph. sergenti apyrase salivary PsSP42 (VR1020-PsSP42 and NTC-PsSP42), empty plasmids (VR1020 and NTC), or PBS (control). All animals were subcutaneously challenged with 2 × 10^7^ late stationary phase of L. tropica along with 0.5 pairs of Ph. sergenti SGH in the right footpad. Eight weeks post challenge, the number of parasites per cell was determined by qRT-PCR from each individual lymph node (six mice per group). Student’s t-test was used for statistical analysis. (* p<0.05, ** p<0.01; ns, nonsignificant). All data are presented as mean ± SD. The data presented here are representative of two independent experiments. (The parasite burden dataset of the second experiment is given in Additional File 9, Fig. S4). [file 13071_2026_7255_MOESM9_ESM.tif]
