# Supplementary material for: Evaluating the effect of immunization with DNA encoding Phlebotomus sergenti apyrase protein (PsSP42) against Leishmania tropica infection in BALB/c mouse model
Source: Parasit Vectors. 2026 Mar 9;19:163. doi: 10.1186/s13071-026-07255-x (PMC13085537; doi:10.1186/s13071-026-07255-x)
Supplement: Supplementary file 3 — Additional file 3: Table S1: Physicochemical parameters of PsSP40, PsSP41, and PsSP42. [file 13071_2026_7255_MOESM3_ESM.docx]

**Table S1: Physicochemical parameters of PsSP40, PsSP41 and PsSP42**

| **Physio-chemical properties** | **PsSP40** | **PsSP41** | **PsSP42** |
| --- | --- | --- | --- |
| **Number of amino acids** | 315 | 295 | 317 |
| **Molecular weight (KD)** | 35.5 | 33.4 | 35.9 |
| **Theoretical PI** | 8.94 | 8.48 | 8.94 |
| **Instability index *** | 15.20 | 17.99 | 16.53 |
| **GRAVY **** | -0.430 | -0.427 | -0.477 |

*****Protein scored below 40 are stable

** The GRAVY score ranges between -2 and 2
