# Supplementary material for: Evaluating the effect of immunization with DNA encoding Phlebotomus sergenti apyrase protein (PsSP42) against Leishmania tropica infection in BALB/c mouse model
Source: Parasit Vectors. 2026 Mar 9;19:163. doi: 10.1186/s13071-026-07255-x (PMC13085537; doi:10.1186/s13071-026-07255-x)
Supplement: Supplementary file 4 — Additional file 4: Table S2: Allergenicity and antigenicity evaluation of PsSP40, PsSP41, and PsSP42. [file 13071_2026_7255_MOESM4_ESM.docx]

**Table S2:** Allergenicity and Antigenicity evaluation of PsSP40, PsSP41 and PsSP42

| **Apyrase proteins** | **AllergenFP v.2.0** | **AllerTop v.1.0** | **ANTIGENpro*** |
| --- | --- | --- | --- |
| **PsSP40** | Probable Non-Allergen | Probable Allergen | 0.83 |
| **PsSP41** | Probable Non-Allergen | Probable Non-Allergen | 0.85 |
| **PsSP42** | Probable Non-Allergen | Probable Non-Allergen | 0.79 |

*Proteins scored over 0.5 are considered as antigen
