# Supplementary material for: Evaluating the effect of immunization with DNA encoding Phlebotomus sergenti apyrase protein (PsSP42) against Leishmania tropica infection in BALB/c mouse model
Source: Parasit Vectors. 2026 Mar 9;19:163. doi: 10.1186/s13071-026-07255-x (PMC13085537; doi:10.1186/s13071-026-07255-x)
Supplement: Supplementary file 6 — Additional file 6: Table S3: Mean ± SD of cytokine production and raw data for each immunized and control groups before L. tropica + SGH challenge. [file 13071_2026_7255_MOESM6_ESM.docx]

**Table S3:** Mean ± SD of cytokine production and raw data for each immunized and control groups before *L. tropica* + SGH challenge.

| **IFN-γ/IL-4**  **Mean ± SD** | **IL-4**  **Mean ± SD** | **IFN-γ**  **Mean ± SD** | **Groups** |
| --- | --- | --- | --- |
| **7.93±2.50** | **11.88±0.46** | **93.96±28.41** | **G1=VR1020-PsSP42** |
| **3.65±1.13** | **10.12±0.45** | **36.50±9.67** | **G2= NTC-PsSP42** |
| **12.0±6.21** | **10.91±0.53** | **129.23±64.06** | **G3=VR1020** |
| **3.23±1.51** | **12.24±0.46** | **39.64±18.89** | **G4=NTC** |
| **2.46±1.23** | **12.15±0.34** | **29.85±15.16** | **G5=PBS** |

| BPS | NTC | VR1020 | NTC-PsSP42 | VR1020-PsSP42 | IFN-γ  , Before challenge response (Raw data) |
| --- | --- | --- | --- | --- | --- |
| 44.19943 | 18.61815 | 214.5013 | 36 | 72.59432 |  |
| 23.30443 | 36.44056 | 129 | 30.07039 | 123.0812 |  |
| 40.25816 | 39 | 113.3868 | 50.33488 | 66.78021 |  |
| 11.62825 | 64.49988 | 60.01859 | 29.6 | 113.3868 |  |
| 29.84757 | 39.63965 | 129.2267 | 36.50132 | 93.96063 | Mean |
| 15.156 | 18.89072 | 64.0637 | 9.671328 | 28.40586 | SD |

| BPS | NTC | VR1020 | NTC-PsSP42 | VR1020-PsSP42 | IL-4  , Before challenge response (Raw data) |
| --- | --- | --- | --- | --- | --- |
| 12.05821 | 12.05821 | 10.6419 | 9.944452 | 11.70155 |  |
| 11.70155 | 12.77648 | 10.29225 | 10.29225 | 11.34659 |  |
| 12.41653 | 11.70155 | 11.34659 | 9.598551 | 12.05821 |  |
| 12.41653 | 12.41653 | 11.34659 | 10.6419 | 12.41653 |  |
| 12.14821 | 12.23819 | 10.90683 | 10.11929 | 11.88072 | Mean |
| 0.342343 | 0.462578 | 0.52747 | 0.448989 | 0.460432 | SD |

| BPS | NTC | VR1020 | NTC-PsSP42 | VR1020-PsSP42 | IFN-γ/ IL-4  , Before challenge response (Raw data) |
| --- | --- | --- | --- | --- | --- |
| 3.665505 | 1.544023 | 20.1563 | 3.620109 | 6.203821 |  |
| 1.991568 | 2.85216 | 12.5337 | 2.921654 | 10.84742 |  |
| 3.242304 | 3.332892 | 9.993029 | 5.244008 | 5.538153 |  |
| 0.936514 | 5.194678 | 5.289571 | 2.781458 | 9.131923 |  |
| 2.458973 | 3.230938 | 11.99315 | 3.641807 | 7.930329 | Mean |
| 1.239042 | 1.511713 | 6.214704 | 1.129357 | 2.493764 | SD |
