# Supplementary material for: Evaluating the effect of immunization with DNA encoding Phlebotomus sergenti apyrase protein (PsSP42) against Leishmania tropica infection in BALB/c mouse model
Source: Parasit Vectors. 2026 Mar 9;19:163. doi: 10.1186/s13071-026-07255-x (PMC13085537; doi:10.1186/s13071-026-07255-x)
Supplement: Supplementary file 7 — Additional file 7: Table S4: Mean ± SD of cytokine production and raw data for each immunized and control groups after challenge with L. tropica + SGH. [file 13071_2026_7255_MOESM7_ESM.docx]

**Table S4:** Mean ± SD of cytokine production and raw data for each immunized and control groups after challenge with *L. tropica* **+** SGH**.**

| **IFN-γ/IL-4**  **Mean ± SD** | **IL-4**  **Mean ± SD** | **IFN-γ**  **Mean ± SD** | **Groups** |
| --- | --- | --- | --- |
| **21.046±15.84** | **43.49±35.04** | **1078.38±1146.89** | **G1=VR1020-PsSP42** |
| **24.04±12.91** | **150.54±63.36** | **3042.73±616.074** | **G2=NTC-PsSP42** |
| **15.058±11.17** | **116.17±136.82** | **1688.23±1652.84** | **G3=VR1020** |
| **12.55±3.40** | **229.08±73.20** | **2756.08±852.24** | **G4=NTC** |
| **10.00±3.03** | **306.66±185.53** | **3240.62±383.27** | **G5=PBS** |

| BPS | NTC | VR1020 | NTC-PsSP42 | VR1020-PsSP42 | IFN-γ  ,After challenge response (Raw data) |
| --- | --- | --- | --- | --- | --- |
| 3291.42 | 3737.657 | 2820.762 | 3016.643 | 585.2401 |  |
| 3488.844 | 3200.323 | 3366.579 | 3196.202 | 1698.217 |  |
| 3446.521 | 2071.604 | 12.93827 | 4179.728 | 259.8008 |  |
| 2567.725 | 2014.753 | 552.6445 | 2609.992 | 26.61626 |  |
| 3408.577 |  |  | 2781.324 | 798.4853 |  |
|  |  |  | 2472.516 | 3101.914 |  |
| 3240.617 | 2756.084 | 1688.231 | 3042.734 | 1078.379 | Mean |
| 383.2715 | 852.236 | 1652.84 | 616.0743 | 1146.882 | SD |

| BPS | NTC | VR1020 | NTC-PsSP42 | VR1020-PsSP42 | IL-4  ,After challenge response (Raw data) |
| --- | --- | --- | --- | --- | --- |
| 96.47337 | 297.4596 | 126.8617 | 165.4284 | 56.4445 |  |
| 257.7188 | 248.501 | 305.7499 | 141.9899 | 35.74374 |  |
| 392.9681 | 125.3123 | 10.6419 | 242.1946 | 17.97116 |  |
| 207.7015 | 245.058 | 21.44539 | 52.82035 | 11.34659 |  |
| 578.4457 |  |  | 120.1691 | 31.9615 |  |
|  |  |  | 180.625 | 107.4634 |  |
| 306.6615 | 229.0827 | 116.1747 | 150.5379 | 43.48848 | Mean |
| 185.5283 | 73.20286 | 136.8257 | 63.35923 | 35.03637 | SD |

| BPS | NTC | VR1020 | NTC-PsSP42 | VR1020-PsSP42 | IFN-γ/IL-4  ,After challenge response (Raw data) |
| --- | --- | --- | --- | --- | --- |
| 9.458103 | 12.56526 | 22.23493 | 18.23534 | 10.36842 |  |
| 13.53741 | 12.87851 | 11.01089 | 22.51007 | 26.61029 |  |
| 8.770486 | 16.53153 | 1.215786 | 17.25772 | 47.51089 |  |
| 12.36257 | 8.221534 | 25.76985 | 49.41262 | 14.45654 |  |
| 5.892647 |  |  | 23.14509 | 2.345749 |  |
|  |  |  | 13.68867 | 24.98272 |  |
| 10.00424 | 12.54921 | 15.05786 | 24.04159 | 21.04577 | Mean |
| 3.031939 | 3.400801 | 11.16898 | 12.91321 | 15.83654 | SD |
