# Supplementary material for: Evaluating the effect of immunization with DNA encoding Phlebotomus sergenti apyrase protein (PsSP42) against Leishmania tropica infection in BALB/c mouse model
Source: Parasit Vectors. 2026 Mar 9;19:163. doi: 10.1186/s13071-026-07255-x (PMC13085537; doi:10.1186/s13071-026-07255-x)
Supplement: Supplementary file 8 — Additional file 8: Table S5: Mean ± SD of Parasite burden and raw data for each immunized and control group after challenge with L. tropica + SGH. [file 13071_2026_7255_MOESM8_ESM.docx]

**Table S5:** Mean ± SD of Parasite burden and raw data for each immunized and control group after challenge with *L. tropica* + SGH.

| **Parasite burden**  **Mean ± SD** | **Groups** |
| --- | --- |
| **1996.57±688.94** | **G1=VR1020-PsSP42** |
| **2474.37±554.43** | **G2=NTC-PsSP42** |
| **3164.14±2004.79** | **G3=VR1020** |
| **10084.52±4740.87** | **G4=NTC** |
| **4028.56±2613.99** | **G5=PBS** |

| BPS | NTC | VR1020 | NTC-PsSP42 | VR1020-PsSP42 | Parasite Burden (Raw data) |
| --- | --- | --- | --- | --- | --- |
| 2573.716 | 16233.76 | 1946.177 | 2277.46 | 2538.582 |  |
| 2118.789 | 4688.354 | 6161.29 | 1980.951 | 2127.031 |  |
| 8532.348 | 10033.69 | 2339.327 | 3557.432 | 1399.128 |  |
| 2895.472 | 9382.284 | 2209.756 | 2220.429 | 1083.703 |  |
| 4022.471 |  |  | 2466.537 | 1905.034 |  |
|  |  |  | 2343.388 | 2925.954 |  |
| 4028.559 | 10084.52 | 3164.138 | 2474.366 | 1996.572 | Mean |
| 2613.993 | 4740.868 | 2004.787 | 554.4299 | 688.9403 | SD |
